# A hypothesized mechanism by which inhaled low-concentration ethanol vapor may bias early entry dynamics of enveloped respiratory viruses

**DOI:** 10.3389/fcimb.2026.1789516

**Published:** 2026-05-06

**Authors:** Yu Wan

**Affiliations:** Harbin Wanyu Technology Co., Ltd., Harbin, China

**Keywords:** enveloped respiratory viruses, ethanol vapor, gas–liquid interface, membrane fluidity, spike conformational dynamics, viral entry efficiency, viral envelope biophysics

## Abstract

Enveloped respiratory viruses rely on conserved biophysical properties of their lipid envelopes for successful host-cell entry, including membrane fluidity, spike conformational mobility, and coordinated fusion activation. These properties impose variation-independent constraints that may serve as potential targets for sequence-agnostic antiviral modulation. Here, a mechanistic hypothesis is proposed in which, under physiologically humid respiratory system conditions, inhalation of low-concentration ethanol vapor may generate a transient, ethanol-enriched microenvironment at airway surface liquid (ASL) and alveolar lining fluid (ALF) interfaces. Rapid vapor–liquid partitioning is hypothesized to permit short-lived interactions between ethanol molecules and viral lipid envelopes during airborne transit or early epithelial contact. Such interactions may transiently increase envelope rigidity, reduce membrane fluidity, constrain spike conformational dynamics, and raise the energetic barrier for membrane fusion, thereby biasing early entry processes against successful infection. This mechanistic framework is grounded in established principles of membrane biophysics, amphiphile–lipid interactions, and diffusion kinetics. It focuses on localized physicochemical modulation at gas–liquid interfaces and does not invoke systemic ethanol exposure, therapeutic dosing, or clinical intervention. In addition, delayed viral entry kinetics arising from altered envelope mechanics may, in principle, modulate the timing of host immune activation, potentially attenuating excessive inflammatory responses. By targeting conserved envelope mechanics rather than sequence-specific viral components, this hypothesis introduces physical microenvironmental modulation as a complementary conceptual domain in antiviral research and provides a foundation for future experimental and computational evaluation of enveloped virus entry dynamics.

## Graphical Abstract

**Figure:**
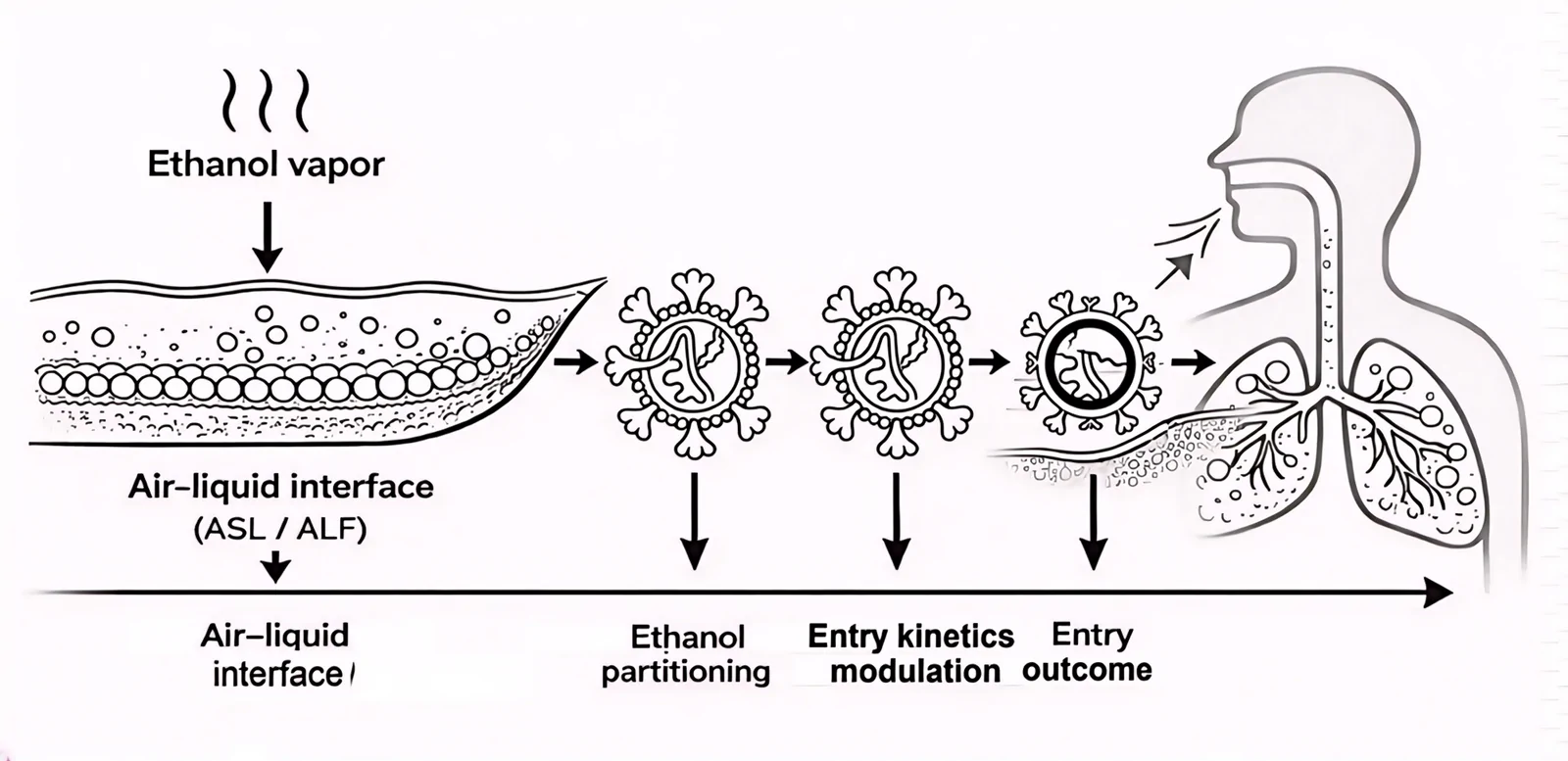
Inhaled ethanol vapor may partition into airway and alveolar surface liquids, transiently enriching the interfacial microenvironment at humid gas–liquid interfaces. As enveloped respiratory viruses traverse this region, ethanol may transiently interact with the viral lipid envelope, potentially modulating envelope mechanical properties and affecting envelope–spike coupling. These physicochemical effects could influence spike conformational dynamics, ACE2 engagement probability, and protease-dependent activation involved in membrane fusion, thereby altering the probability of productive infection.

## 1 Introduction

The COVID-19 pandemic exposed the world’s significant vulnerability to rapidly spreading enveloped respiratory viruses, underscoring the limitations of existing antiviral preparedness frameworks (Perelson and Ke, 2021). While vaccines and antiviral drugs have substantially reduced disease severity and mortality, their effectiveness continues to face challenges such as rapid viral mutation, delays in production and distribution, and uneven global accessibility. These persistent constraints highlight an urgent need for complementary antiviral paradigms—those characterized by anti-mutational properties, rapid scalability, and independence from immune initiation mechanisms.

Ethanol is a low-cost, globally accessible small-molecule amphiphile with well-defined biochemical and physicochemical properties. The sensitivity of enveloped viruses to alcohol-mediated physicochemical perturbation in surface disinfection and liquid-phase exposure scenarios is extensively documented (Chernomordik and Kozlov, 2008; Harrison, 2008). Beyond these applications, inhaled ethanol has historically been used only in limited clinical settings—such as pulmonary edema treatment and neonatal respiratory support—with no antiviral intent. Despite this long history of use, the potential mechanisms by which ethanol might modulate viral infectivity within the human respiratory system remain relatively understudied. This gap primarily stems from the common assumption that the ethanol concentrations achievable via inhalation are too low, the exposure time too brief, or the spatial distribution too widespread to exert a significant effect on the viral invasion process (Perelson and Ke, 2021).

Notably, the successful invasion of host cells by enveloped viruses, including SARS-CoV-2, is not solely dependent on receptor recognition or sequence-specific molecular interactions. This process is critically contingent upon the biophysical properties of the viral lipid envelope, including membrane fluidity, deformability, curvature adaptability, and the cooperative proteolytic activation of the fusion machinery (Wanner et al., 1996; Boulant et al., 2015). These requirements impose conserved biophysical constraints that largely remain invariant across viral variants and lineages. Consequently, enveloped viruses may be particularly susceptible to physicochemical perturbations that alter lipid order and membrane mechanics, even when such perturbations occur below levels that cause overt membrane disruption.

At sub-lytic and non-disruptive concentrations, ethanol has been shown to alter membrane packing density, reduce lipid lateral diffusion (Crank, 1975), and modify the functional dynamics of membrane-associated proteins without causing significant structural damage (Harrison, 2008; West, 2016). These effects have been observed across various lipid bilayer systems. These properties are particularly relevant to cholesterol-containing membranes, which closely resemble the physicochemical composition of the viral envelope. These established characteristics provide a solid biophysical rationale for considering whether transient ethanol exposure might regulate viral entry efficiency through mechanical and energetic pathways rather than direct biochemical inactivation.

This study hypothesizes that the inhalation of low-concentration ethanol vapor creates a transient, ethanol-enriched microenvironment at the respiratory system’s air–liquid interface. Under physiologically humid conditions, gas-phase ethanol may rapidly partition into the airway surface liquid and alveolar lining fluid layers, enabling brief interactions with viral particles during their transit through the respiratory tract (Hsia et al., 2016). In the conducting airways, this interface is formed by the Airway Surface Liquid (ASL), whereas in the distal lung the Alveolar Lining Fluid (ALF) covering the alveolar epithelium provides a functionally analogous air–liquid interface. These microenvironments extend from the conducting airways to the distal alveolar air–liquid interfaces, an anatomical region critically associated with severe viral pneumonia and pathological amplification.

In these localized fluid microenvironments, ethanol molecules may briefly integrate into the viral lipid envelope, restricting spike protein conformational dynamics, reducing the effective accessibility of the S1/S2 activation domain, and increasing the energy barrier for membrane fusion at the earliest stage of host cell entry (Wanner et al., 1996; Boulant et al., 2015). Crucially, this hypothesis does not involve systemic ethanol exposure, therapeutic dosages, or clinical intervention.

**Figure 1:**
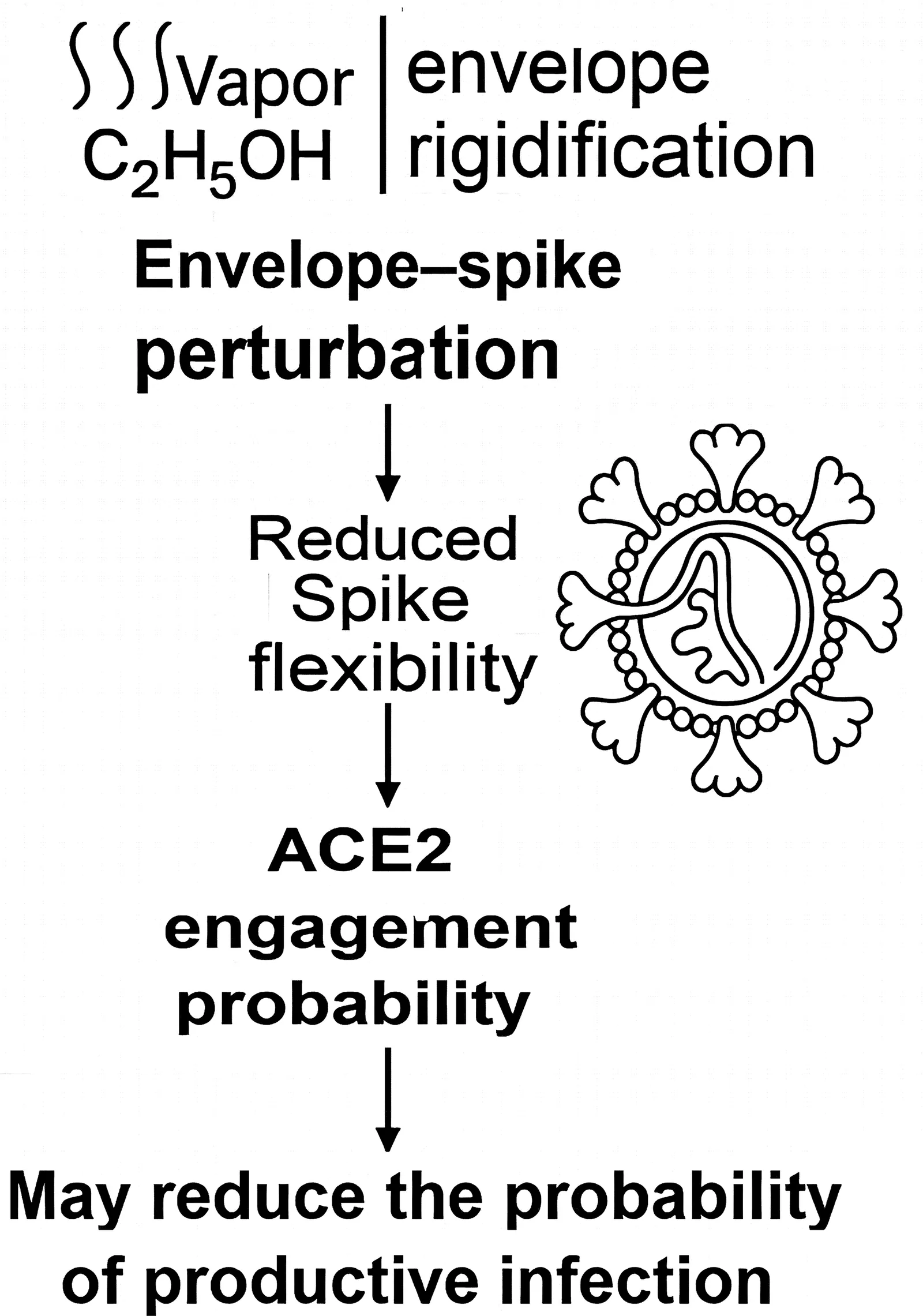
Conceptual model of ethanol vapor effects on viral envelope mechanics and entry dynamics. Exposure to ethanol vapor (C_2_H_5_OH) is proposed to modulate viral envelope mechanics and perturb envelope–spike dynamics. These biophysical alterations may influence spike conformational behavior and ACE2 engagement probability, thereby altering the likelihood of productive infection. This schematic represents a mechanistic hypothesis. Elements are not drawn to scale, and no direct biochemical interference of host receptors or proteases is implied.

This research offers no clinical evidence or therapeutic recommendations; rather, it proposes a general mechanistic framework based on principles of membrane biophysics, physicochemical partitioning, and diffusion kinetics. Its objective is to explore how altering the properties of the early invasion process—by momentarily altering the mechanical state of the viral envelope at the respiratory gas-liquid interface—might bias early viral entry efficiency (**Figure 1**). This perspective offers a complement to existing antiviral strategies by targeting the conserved physical properties of the viral particle instead of sequence-specific molecular motifs.

The respiratory system is a highly heterogeneous anatomical and physicochemical environment, characterized by significant regional differences in airway and alveolar surface geometry, epithelial arrangement, airflow velocity, and surface liquid composition. Classic morphometric analysis and subsequent pathological studies confirm that these structural gradients play a key role in shaping gas transport, particle deposition, and early pathogen-host interaction (Weibel, 1963; Hogg and Timens, 2009). The distal alveolar region, in particular, is the primary site where pathological processes—including viral replication, epithelial damage, inflammatory infiltration, and gas exchange impairment—converge, factors that collectively drive the severe progression and elevated mortality rates associated with viral pneumonia.

Consistent with this view, experimental and modeling studies on the behavior of inhaled particles demonstrate significant differences in deposition efficiency and residence time between the nasal cavity, conducting airways, and distal alveolar regions, depending on particle size, airflow patterns, and airway structure (Anderson et al., 1990; Geiser and Kreyling, 2010). These spatially resolved deposition processes define the physical environments where inhaled viral particles first encounter the airway surface liquid and alveolar lining fluid before contacting the epithelial cells. Therefore, even minor perturbations to the viral envelope mechanics occurring at the alveolar air-liquid interface may have a disproportionate pathological significance by influencing viral entry at the anatomical site most closely associated with respiratory failure.

It is critical to state that the following mechanism is presented strictly as a non-therapeutic, conceptual framework. This hypothesis does not involve human or animal studies, nor does it establish safety, efficacy, or clinical protocols. The discussion is meticulously constrained by physicochemical boundaries: focusing only on interfacial ethanol vapor interactions characterized by transient local enrichment at the air–liquid interface and their effects on conserved viral envelope mechanics. This work aims only to define a falsifiable mechanistic space where a physical perturbation that is less sensitive to viral sequence variation could theoretically influence early viral entry kinetics.

During the early phase of the COVID-19 pandemic, non-systematic real-world observations provided an unexpected conceptual trigger for the present hypothesis. In that public-health context, the absence of rapid relief under routine management prompted a mechanistic question rather than an efficacy claim: whether a localized, transient physicochemical perturbation at humid respiratory gas–liquid interfaces could bias virus-associated processes without invoking systemic exposure or direct virucidal dosing.

Notably, it was the presence of unexpected macroscopic observations—despite the absence of controls, reproducibility, or systematic validation—that catalyzed subsequent mechanistic reasoning and theoretical development; had no such perceptible phenomenon been noted, there would have been little impetus to pursue deeper, non-intuitive mechanisms. These observations are therefore presented solely as historical context motivating hypothesis generation, not as experimental, preclinical, or clinical evidence, and not as a basis for any recommendation, exposure specification, or causal inference.

Experimental and computational studies have shown that short-chain alcohols, including ethanol, can interact with lipid bilayers at sub-solubilizing concentrations by partitioning into the headgroup region and perturbing lipid packing, lateral pressure profiles, and membrane elasticity (Rowe, 1987; Cantor, 1997; Feller et al., 2002; Ly and Longo, 2004; Tierney et al., 2005; Terama et al., 2008; Ingólfsson and Andersen, 2011). Such perturbations can influence bilayer thickness, fluidity, and bending rigidity without necessarily causing membrane dissolution (Evans and Rawicz, 1990; Seifert, 1997; Nagle and Tristram-Nagle, 2000). Because membrane mechanics and lipid composition play key roles in viral membrane fusion and entry processes (Harrison, 2008; Lorizate and Kräusslich, 2011; Kozlov and Chernomordik, 2015), these physicochemical effects provide a plausible biophysical basis for exploring whether modest ethanol exposure could influence early virus–host membrane interactions. The author first proposed and systematically described the biophysical mechanism and conceptual framework of low-concentration ethanol vapor intervention in early 2020 (Wan, 2020). The present study develops the theoretical and mechanistic basis for that earlier conceptual framework.

## 2 Mechanistic framework

### 2.1 Respiratory gas–liquid interfaces and viral deposition

In this manuscript, the term “respiratory” refers to the entire respiratory tract, encompassing both the conducting airways and the distal alveolar regions. These compartments are characterized by humid gas–liquid interfaces formed by the airway surface liquid (ASL) in the conducting airways and the alveolar lining fluid (ALF) covering the alveolar epithelium. These interface-proximal liquid layers represent the first physicochemical environment encountered by inhaled aerosols, particles, and viral virions before epithelial contact.

The physicochemical environment at respiratory air–liquid interfaces is highly dynamic. Local ethanol partitioning would be influenced not only by equilibrium gas–liquid partitioning but also by environmental and physiological factors including humidity, temperature, respiratory airflow, epithelial ion transport, and the continuous renewal of airway surface liquid through mucociliary clearance (Bustamante-Marin and Ostrowski, 2017). Consequently, any local interfacial variation at membrane-proximal interfaces should be considered transient and spatially heterogeneous rather than a static equilibrium condition. Gas–liquid partitioning of small volatile molecules typically occurs on rapid timescales relative to many transport processes in respiratory surface liquids.

Because enveloped respiratory viruses must traverse these surface liquids prior to host-cell entry, the physicochemical properties of ASL and ALF may influence early infection dynamics and the residence time of virions within interface-proximal compartments. These considerations raise the possibility that transient perturbations at respiratory gas–liquid interfaces may influence early virus–host interactions through effects on viral membrane mechanics.

Although a full predictive model is beyond the scope of the present study, the proposed enrichment problem can be expressed in semi-quantitative form as:

The following formulation is intended as a conceptual representation of coupled physicochemical factors rather than a predictive transport model.


$$C{\_}_{local}(t)\approx C{\_}_{gas}H\text{Φ}(D,\delta ,t{\_}_{res})K_{\_int}K{\_}_{mem}$$

Here *C_gas_* denotes the gas-phase ethanol concentration and *H* the gas–liquid partition coefficient term. Φ(D, δ, t_res) represents a transient transport factor determined by diffusivity D, characteristic interfacial path length δ, and local residence time t_res. K_int denotes a potential local interfacial enrichment factor, and K_mem represents partitioning into membrane-proximal regions.

Under this formulation, the enrichment ratio may be written as:


$$E=\frac{C_{\_local}/}{C_{\_bulk}}$$

This expression does not establish a numerical local interfacial variation but identifies the coupled physical variables that could govern deviations between local and bulk-equivalent exposure in respiratory interfacial microenvironments. A conceptual comparison is summarized in **Table 1**.

**Table 1:** Conceptual comparison of respiratory interfacial microenvironments relevant to ethanol partitioning.

Anatomical region	Interfacial liquid	Approximate path length (d)	Dominant clearance process	Implication for local ethanol distribution
Conducting airways	Airway surface liquid (ASL)	Relatively thick (~5–10 μm)	Rapid mucociliary clearance	Local deviations from bulk-equivalent exposure likely transient
Distal alveoli	Alveolar lining fluid (ALF)	Ultra-thin (<1 μm)	Surfactant turnover and diffusion	Gas–liquid partitioning may approach near-equilibrium conditions

This table summarizes representative respiratory interfacial environments that may influence ethanol partitioning. The comparison is conceptual and intended to illustrate differences in transport distance and clearance processes rather than to provide quantitative predictions.

### 2.2 Biophysical constraints of enveloped viral entry

Enveloped respiratory viruses, including SARS-CoV-2, possess lipid-rich envelopes derived from host cell membranes. Viral infectivity depends on coordinated interactions between viral fusion proteins and the physical properties of the lipid envelope, including membrane fluidity, deformability, and curvature adaptability. Successful entry generally involves receptor engagement followed by protease-mediated activation of the spike protein at the S1/S2 cleavage site, which initiates membrane fusion processes enabling delivery of the viral genome into host cells (Wanner et al., 1996; Boulant et al., 2015).

These processes impose fundamental biophysical constraints on the viral envelope. Membrane fusion typically requires highly localized membrane curvature generation, involving intermediates such as the hemifusion stalk and subsequent fusion pore formation. The energetic barriers associated with these transitions are influenced not only by conformational changes of viral fusion proteins but also by the intrinsic mechanical properties of the surrounding lipid bilayer.

From a membrane biophysics perspective, lipid bilayers can be described as elastic surfaces characterized by parameters such as bending rigidity, lateral tension, and curvature elasticity. Typical phospholipid bilayers exhibit bending rigidities on the order of 10–30 k_BT, providing a useful conceptual reference for the energetic scale associated with membrane deformation. In addition to global elastic properties, the energetic landscape of membrane remodeling may also depend on the internal lateral pressure profile across the bilayer thickness, which reflects the balance of forces acting within different regions of the lipid membrane.

Small amphiphilic molecules present in the surrounding environment may influence these physical properties. Molecules such as ethanol are known to partition into lipid membranes and preferentially localize near membrane–water interfaces. Experimental and computational studies have shown that such partitioning can modify lipid packing, lateral pressure distributions, and membrane elasticity under certain conditions. These physicochemical perturbations may occur at concentrations that do not lead to membrane solubilization.

Under such circumstances, alterations in lipid organization or interfacial stress distribution could influence the energetic landscape associated with membrane fusion intermediates. For example, changes in membrane packing or curvature elasticity may affect the energetic cost of forming highly curved structures such as hemifusion stalks. In principle, such effects could modify the balance between the energy released by viral fusion protein conformational changes and the mechanical work required to deform the lipid bilayer during membrane merger.

Importantly, these membrane mechanical constraints arise from lipid–protein assemblies rather than directly from viral genome sequence. As a result, the physical requirements governing membrane fusion are expected to be comparatively less sensitive to genetic variation among viral variants than processes determined solely by protein structure. Consequently, perturbations affecting lipid organization or membrane elasticity may influence the energetic landscape governing membrane fusion processes, even without directly inactivating viral particles. A conceptual comparison of representative membrane systems and their mechanical properties is summarized in **Table 2**.

**Table 2:** Conceptual comparison of membrane mechanical properties and potential responses to ethanol perturbation.

Membrane system	Typical bending rigidity (κ)	Structural reinforcement	Potential response to ethanol perturbation	Possible biophysical implication
Viral envelope (nanoscale membrane)	~10–20 kBT (representative literature range for nanoscale viral membranes)	None (free membrane vesicle)	Small perturbations in lipid packing may influence membrane curvature elasticity	May alter the energetic landscape of membrane fusion intermediates
Host plasma membrane	~20–40 kBT (composition dependent)	Cytoskeletal coupling and cholesterol enrichment	Structural reinforcement may buffer small membrane perturbations	Overall membrane mechanical stability likely maintained
Model phospholipid bilayers	~10–30 kBT (composition dependent)	Experimental system dependent	Reported to respond to short-chain alcohol partitioning	Useful reference system for studying ethanol–membrane interactions

Values represent typical ranges reported in membrane biophysics studies and are provided for conceptual comparison only. Descriptions of ethanol responses are qualitative and do not constitute quantitative predictions.

### 2.3 Interfacial partitioning and membrane perturbation by ethanol

Volatile molecules present in the gas phase may transiently partition into humid respiratory gas–liquid interfaces such as the airway surface liquid (ASL) and alveolar lining fluid (ALF). As a conceptual reference, Henry’s law relates the equilibrium dissolved concentration of a volatile molecule to its gas-phase partial pressure according to C = k_H P. However, the resulting bulk liquid concentration should not be directly interpreted as the effective concentration experienced at viral membrane interfaces. Local exposure may instead depend on additional physicochemical factors, including interfacial partitioning behavior, membrane–water boundary localization, lipid affinity, and transient interfacial accumulation. Gas–liquid partitioning of small volatile molecules typically occurs on rapid timescales, suggesting that such equilibration processes may proceed rapidly relative to early virus–host contact events at respiratory interfaces. Experimental measurements and molecular simulations have shown that ethanol molecules can accumulate preferentially near membrane–water interfaces and may influence lipid packing, lateral pressure distributions, and membrane elasticity (Rowe, 1987; Cantor, 1997; Feller et al., 2002; Ly and Longo, 2004; Tierney et al., 2005; Terama et al., 2008; Ingólfsson and Andersen, 2011). Importantly, such physicochemical perturbations may occur at concentrations below those required for membrane solubilization.

The mechanical responses of viral envelopes and host-cell membranes to these perturbations may differ due to several structural factors. Mammalian plasma membranes typically contain significant levels of cholesterol, which promotes liquid-ordered lipid phases associated with tighter lipid packing and reduced penetration of small amphiphilic molecules. In addition, the host plasma membrane is mechanically coupled to the cortical actin cytoskeleton, which can provide structural reinforcement and damping against transient fluctuations in membrane tension.

Although viral envelopes originate from host-cell membranes, they differ in several physical aspects, including nanoscale curvature, absence of cytoskeletal coupling, and vesicle-like geometry. These features may influence how viral membranes respond to external physicochemical perturbations.

Within the classical Helfrich framework of membrane elasticity, the energetic cost of membrane deformation depends on parameters such as bending rigidity and membrane curvature. Typical phospholipid bilayers exhibit bending moduli on the order of ~10–30 k_BT. For nanoscale vesicles with radii on the order of tens of nanometers (~50–100 nm), this corresponds to membrane deformation energies in the hundreds of k_BT range.

Because viral particles are orders of magnitude smaller than host cells, their envelopes operate under substantially stronger curvature constraints. Under such mechanical regimes, even modest perturbations in membrane elastic properties could influence the energetic landscape associated with membrane fusion intermediates.

While such effects would not necessarily lead to direct viral inactivation, they may alter the balance between protein-driven fusion forces and the mechanical work required for membrane deformation during viral entry.

A simplified conceptual framework can help illustrate the physicochemical boundary conditions governing ethanol exposure at respiratory gas–liquid interfaces, without implying quantitative predictions.

Under equilibrium assumptions, the dissolved ethanol concentration in airway surface liquid can be approximated by C = k_H P, where P represents the gas-phase partial pressure.

For illustrative purposes, ethanol vapor concentrations in the ~10³ ppm range correspond to partial pressures on the order of ~10^-^³ atm. Using typical Henry’s law constants reported for ethanol in aqueous systems, such equilibrium partitioning could theoretically generate ethanol concentrations spanning the high-millimolar regime in bulk liquid phases.

Here C_local represents the effective ethanol exposure at membrane-proximal interfacial regions rather than the actual ethanol concentration within the lipid bilayer.

Importantly, these simplified estimates should not be interpreted as direct predictors of membrane-level exposure. Local ethanol concentrations or mole fractions within viral envelopes or membrane microenvironments may differ substantially due to interfacial enrichment, lipid affinity, and dynamic transport processes at respiratory interfaces. Consequently, the present analysis should be viewed as a conceptual biophysical framework intended to explore potential membrane-level mechanisms rather than as a quantitative prediction of antiviral efficacy.

## 3 Integrated multi-layer mechanistic model

A core distinction central to the current hypothesis is the differentiation between viral infectivity and viral entry efficiency. While often conflated in antiviral research, these concepts represent fundamentally distinct biological and biophysical processes. In experimental, clinical, and epidemiological contexts, viral infectivity is typically operationally viewed as a quasi-binary outcome: a productive entry either occurs or does not. In contrast, viral entry efficiency describes a probabilistic, multi-step process that determines the likelihood of a single virion successfully completing host cell entry under specific physicochemical and biological conditions. Crucially, subtle perturbations in early entry efficiency can have a disproportionate downstream impact on viral amplification kinetics, even in the absence of complete viral inactivation or structural disruption.

The hypothesis presented here specifically targets viral entry efficiency, rather than the destruction or loss of structural integrity of the viral particle. Ethanol vapor-mediated modulation of the viral envelope does not directly destroy or inactivate the virion. Instead, the mechanism operates by reducing the probability that the coordinated sequence of events required for successful viral entry—including attachment, receptor binding, proteolytic activation, membrane deformation, and fusion—will occur within the relevant temporal window. Because early infection dynamics are highly sensitive to the initial entry rate, even a modest reduction in entry probability can significantly attenuate downstream viral replication kinetics and population-level amplification, without relying on a deterministic inhibitory mechanism.

**Figure 2:**
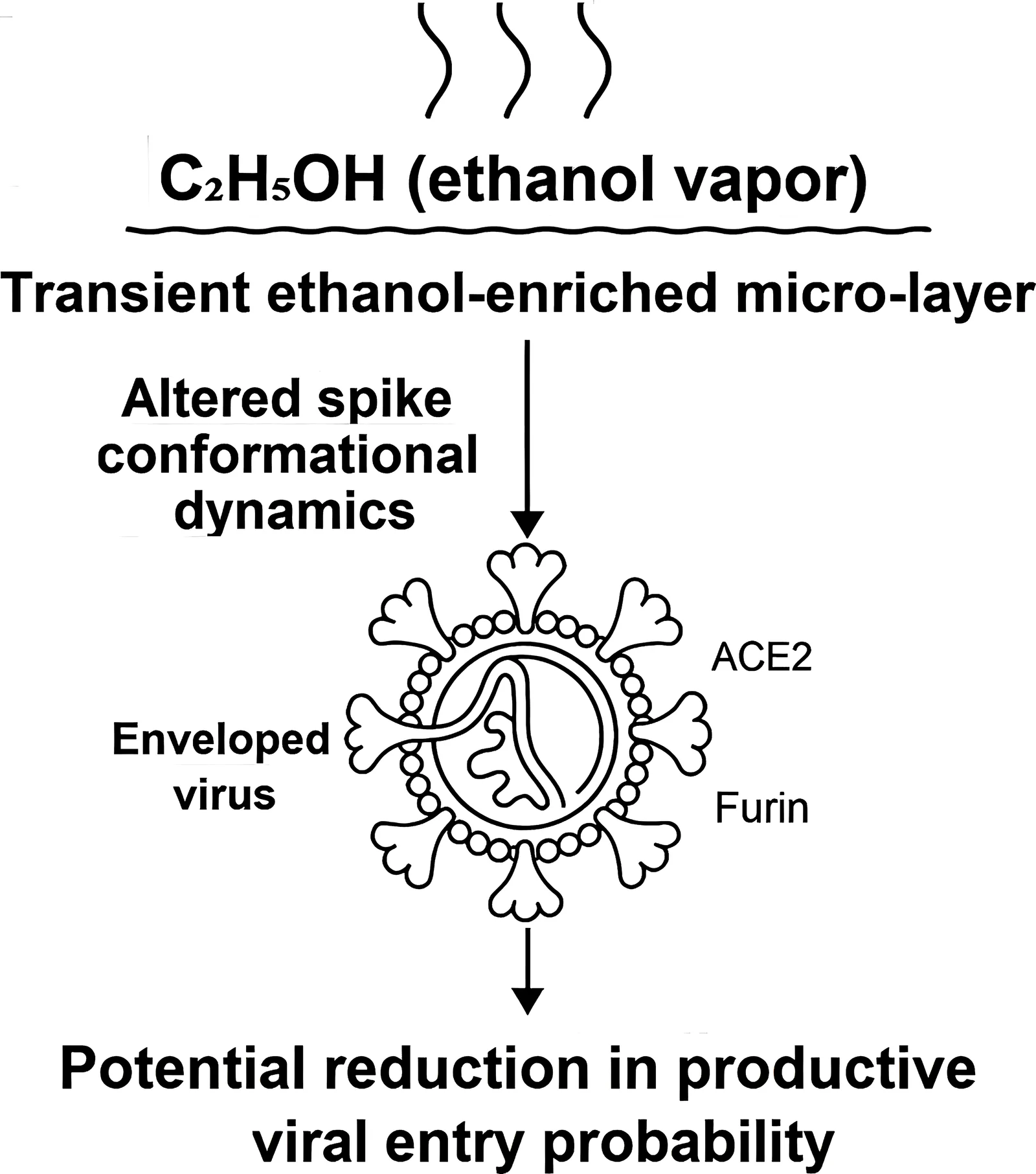
Conceptual model of ethanol vapor–mediated modulation of viral entry. Inhaled ethanol vapor (C_2_H_5_OH) may transiently partition at respiratory gas–liquid interfaces and interact with the airway surface liquid (ASL) and alveolar lining fluid (ALF). Within such humid interfacial environments, virions traversing the air–liquid boundary may encounter localized physicochemical conditions that differ from bulk exposure. Under these conditions, ethanol molecules could influence viral envelope mechanical properties, including membrane elasticity and spike conformational mobility. Such perturbations may, in principle, influence the probability of productive ACE2 engagement and subsequent host protease–dependent activation (e.g., furin-mediated cleavage), thereby potentially modulating viral entry dynamics.

The distribution of ethanol vapor within the respiratory system is governed by a confluence of diffusion kinetics, airflow patterns, and gas-liquid partitioning at the respiratory interface. Unlike systemic delivery, gas-phase exposure focuses on spatial localization and transient dynamics rather than sustained bulk concentration. Upon inhalation, ethanol vapor diffuses rapidly down concentration gradients throughout the respiratory system tract, encountering the air-liquid interface at multiple anatomical sites, including the nasal cavity, trachea, bronchi, distal airways, and alveolar regions. The rapid gas-liquid exchange allows for the transient enrichment of ethanol molecules in the surface liquid, while continuous airflow, surface liquid renewal, and clearance mechanisms limit its long-term accumulation in deeper tissues. Within this framework, the mechanism can be conceptualized as an integrated Multi-Layer Membrane Modulation Model. Transient physicochemical perturbations at the humid air-liquid interface propagate across multiple spatio-temporal scales, thereby influencing viral entry kinetics. Given its high vapor pressure and small molecular size, ethanol vapor diffuses rapidly upon inhalation. At the physiologically humid respiratory interface, ethanol preferentially partitions into the surface liquid layer, forming a transient but functionally relevant ethanol microenvironment that inhaled viral particles must traverse before contacting epithelial cells (**Figure 2**). These interfacial zones constitute the initial contact point for the interaction between the viral particle and ethanol molecules within the respiratory system tract.

### 3.1 Layer 1: formation of the ethanol-enriched surface liquid microlayer

**Figure 3:**
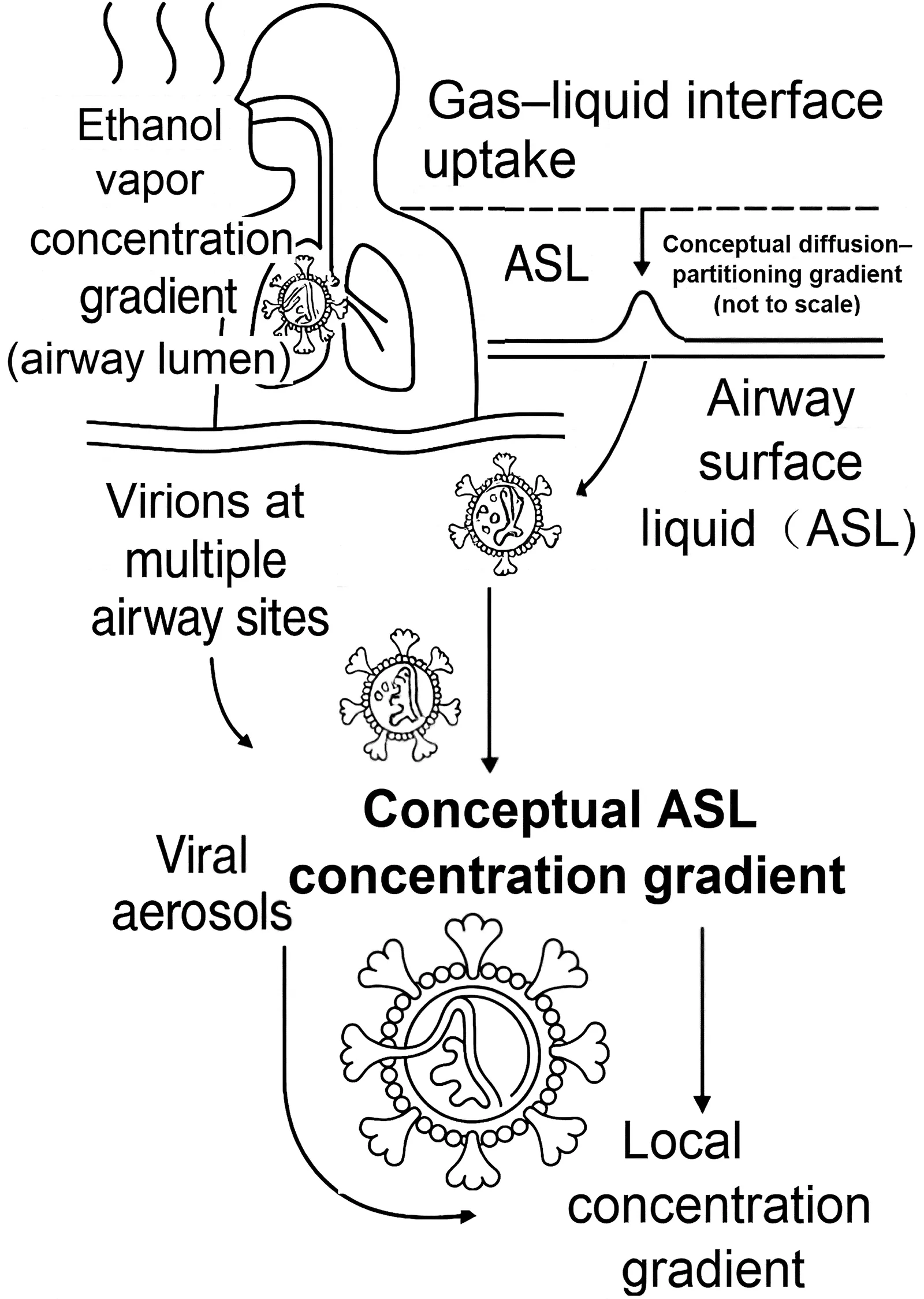
Conceptual diffusion–partitioning model of inhaled ethanol vapor in the respiratory tract. This schematic illustrates a conceptual diffusion and gas–liquid partitioning framework by which inhaled ethanol vapor may transiently associate with airway and alveolar gas–liquid interfaces. The dashed arrow indicates a qualitative diffusion–partitioning gradient shown for conceptual purposes only and not to scale. This figure does not represent quantitative concentration profiles, predictive kinetics, or time-resolved viral population dynamics but provides mechanistic context for localized ethanol–virus interactions across respiratory regions.

The humid respiratory surface favors the partitioning of ethanol from the gas phase into the surface liquid phase. In the conducting airways, this process occurs within the Airway Surface Liquid (ASL); in the distal lung, the Alveolar Lining Fluid (ALF) provides a functionally analogous microenvironment. Diffusion kinetics suggest that local interfacial variation in these surface liquid compartments can occur rapidly post-inhalation, generating a transient local concentration distinct from the bulk air level (**Figure 3**). This enrichment is spatially localized and temporally constrained, reflecting the dynamic changes of continuous airflow, ASL and ALF renewal, mucociliary transport, and alveolar fluid homeostasis. Consequently, the ethanol-enriched surface liquid microlayer is metastable rather than persistent, but remains functionally relevant during the viral particle’s early contact with the respiratory system interface.

### 3.2 Layer 2: biophysical effects on the viral envelope

**Figure 4:**
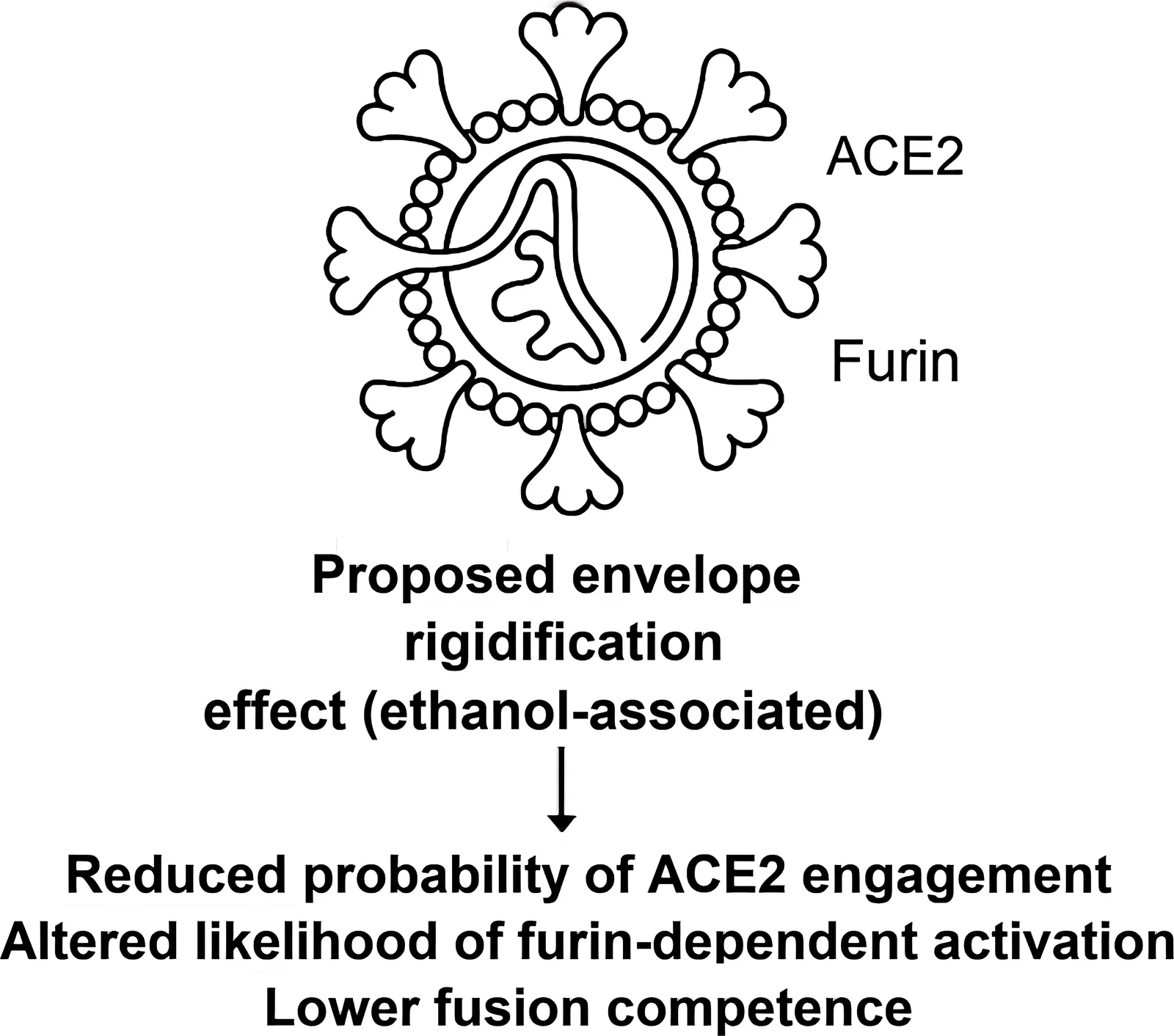
Conceptual model of ethanol-associated modulation of viral envelope mechanics and its functional implications. Ethanol-associated perturbations of viral envelope mechanics are proposed to constrain spike protein dynamics, potentially influencing ACE2 engagement and furin-dependent activation. These combined biophysical effects may alter viral fusion competence during early stages of host cell entry.

As the viral particle enters the ethanol-enriched interfacial microenvironment, ethanol molecules may transiently partition into the viral lipid bilayer. This intercalation modifies the local lipid packing structure, inhibits lateral lipid diffusion, and simultaneously modifies membrane stiffness and elastic properties. These changes raise the energy barrier for the membrane curvature transitions required during viral fusion, shifting the viral envelope from a highly flexible, deformable structure toward a more viscoelastic and mechanically constrained shell (**Figure 4**). These alterations do not imply the destruction of the viral particle, but rather limit the range of membrane shape changes that can occur during invasion.

### 3.3 Layer 3: conformational constraints on spike protein dynamics

Envelope stiffening alters the mechanical coupling between the viral lipid bilayer and embedded spike proteins, thereby constraining the conformational mobility required for rapid spike reorientation and cooperative engagement during entry.

In combination with envelope stiffening (Layer 2), constrained spike dynamics (Layer 3) are hypothesized to increase the kinetic energy barrier required for rapid, coordinated membrane apposition necessary for membrane fusion or clathrin-mediated endocytosis. Rather than preventing entry outright, this elevated barrier is expected to delay early entry kinetics, ultimately reducing the overall rate of successful viral entry events.

### 3.4 Layer 4: reduced accessibility of the S1/S2 cleavage and activation site

**Figure 5:**
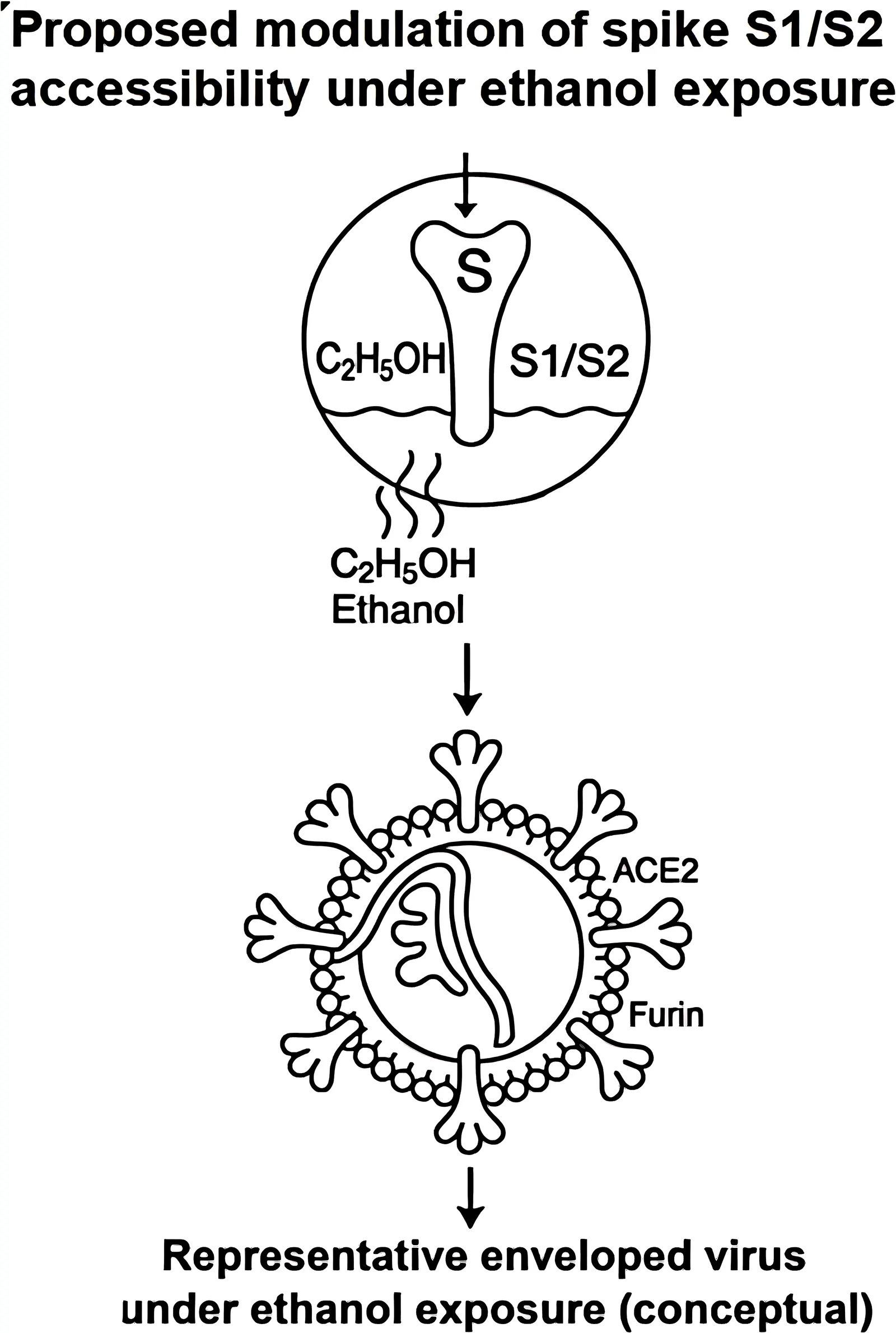
Proposed perturbation of spike S1/S2 region accessibility associated with ethanol. This conceptual illustration depicts a proposed mechanism by which ethanol-associated changes in viral envelope mechanics may bias the geometric orientation and conformational accessibility of the Spike S1/S2 region. Such perturbations are hypothesized to influence the likelihood of furin-dependent activation and subsequent membrane fusion competence during early stages of host cell entry. No direct chemical interaction or virus-specific effects are implied.

Successful membrane fusion in SARS-CoV-2 and many other enveloped viruses requires protease-mediated pre-activation at the S1/S2 cleavage site. Envelope stiffening and altered spike protein orientation reduce the geometric accessibility of this region, thereby lowering the efficiency of Furin-mediated cleavage activation (**Figure 5**). Since S1/S2 pre-activation is a critical checkpoint rather than a terminal step, even a partial reduction in its efficiency can significantly decrease the overall invasion probability without requiring near-complete reduction or irreversible structural damage.

### 3.5 Layer 5: system-level impact on early infection kinetics

**Figure 6:**
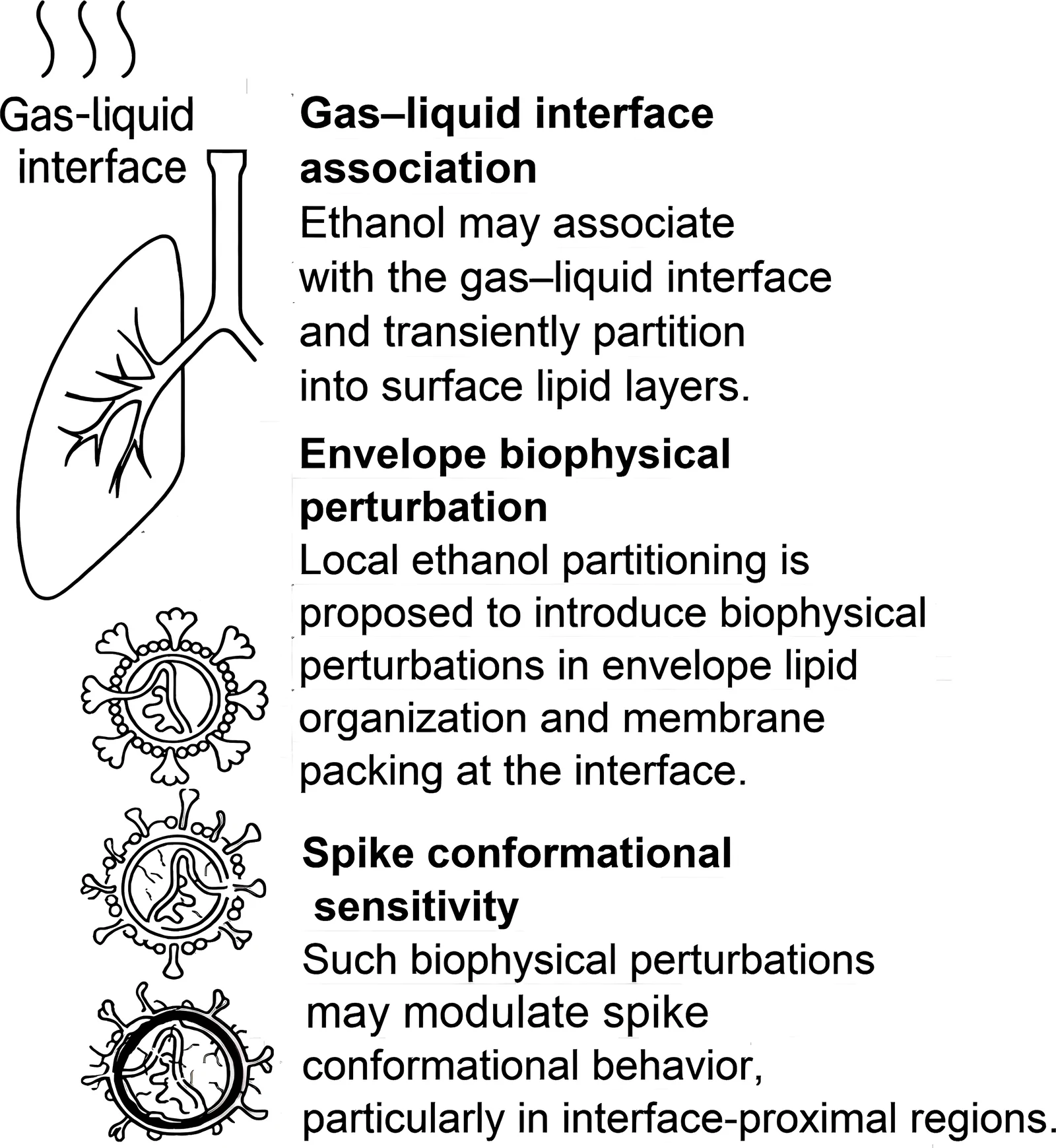
Conceptual overview of system-level probabilistic bias on early viral entry. This schematic illustrates how multiple localized and transient ethanol–virus interactions at respiratory gas–liquid interfaces may collectively bias the probability of successful viral entry. Rather than depicting defined temporal kinetics or cumulative viral damage, the figure illustrates a system-level probabilistic shift in entry outcomes arising from distributed biophysical perturbations.

These multi-layered perturbations collectively create a kinetic bottleneck, significantly slowing the rate at which viral particles successfully enter host cells. Under undisturbed conditions, early infection typically follows a synchronized exponential growth pattern driven by coordinated fusion events across the viral population. Ethanol-induced envelope stiffening, restricted spike protein dynamics, and reduced S1/S2 priming efficiency all lower the probability of successful fusion, introducing heterogeneity and temporal dispersion into early viral entry events (**Figure 6**).

This kinetic delay effectively reduces the rate of productive viral entry but also widens the temporal window available for the innate immune response while limiting the potential for rapid downstream amplification.

It is important to note that, during normal respiration, the alveolar air-liquid interface is not uniformly engaged in gas exchange. Under physiological conditions, only a fraction of the alveolar surface area is involved in active gas exchange at any given moment, reflecting regional differences in ventilation, perfusion, and alveolar recruitment. Consequently, vapor-interface interactions within the distal lung tissue are inherently heterogeneous and probabilistic in nature, rather than spatially uniform. This heterogeneity implies that the viral exposure to interfacial physicochemical perturbation is achieved through a distributed mechanism across the alveolar population.

Given that severity and mortality are primarily dictated by viral replication and damage within the alveolar compartment, even a minor kinetic delay occurring at the alveolar air-liquid interface can have disproportionate pathological significance.

The proposed multi-layer framework is not intended as a set of independent or sequentially separate effects, but as an integrated mechanistic system where a perturbation introduced at one layer propagates to adjacent spatial and temporal scales. Each layer modifies the boundary conditions for the subsequent layer, leading to a cumulative kinetic consequence that cannot be fully captured by considering any single component in isolation.

In summary, these five layers of modulation—surface liquid microlayer formation in the ASL/ALF, envelope stiffening, restricted spike dynamics, attenuated S1/S2 priming, and slowed viral entry kinetics—together constitute an integrated biophysical mechanism by which ethanol vapor may influence the entry dynamics of enveloped respiratory viruses. These interactions act upon the conserved physicochemical properties of the viral envelope rather than variable sequence motifs, establishing a unified framework that is less sensitive to viral sequence variation for modulating the viral early entry process. Importantly, this model remains probabilistic rather than deterministic, emphasizing rate modulation and boundary condition reshaping rather than a binary switch effect. By integrating interface physics, membrane mechanics, protein dynamics, and system-level kinetics into a single conceptual framework, the hypothesis provides a coherent basis for experimentally verifiable predictions while remaining neutral regarding clinical efficacy or intervention strategies.

## 4 Modulation of biological membrane structure and function by ethanol

This section examines the functional implications of envelope-level regulation under clearly defined physicochemical and biological boundary conditions. The focus lies on probabilistic alterations in viral entry behavior rather than direct, deterministic antiviral effects or irreversible structural damage. Indeed, the viral fusion process is highly dependent on membrane fluidity—a dependency that is critical because only through this fluidity can the high-energy coordination required for viral invasion be achieved. Therefore, it is hypothesized that microscale perturbations caused by low-concentration ethanol on membrane fluidity may lead to functional constraints in viral entry processes. Although host cells rely on membrane structures for activities such as endocytosis and signal transduction, their membrane structures are more robust and can tolerate such perturbations without substantial functional disruption.

### 4.1 Mechanistic boundary conditions of envelope-level modulation

Before discussing downstream biological effects, the mechanistic boundary conditions for the interaction between ethanol vapor and the viral particle must be clearly established. Within this framework, ethanol is not acting as a virucidal solvent, disinfectant, or chemical inactivator, but as a transient physicochemical modulator acting at the air-liquid and liquid-membrane interfaces. The proposed interaction occurs at low-concentration, non-disruptive local environments, and within a short temporal window insufficient to cause envelope lysis or irreversible structural damage.

Under these boundary conditions, ethanol molecules may transiently partition into the viral lipid envelope as the viral particle traverses the ethanol-enriched surface liquid microlayer (at respiratory air-liquid interfaces such as the ASL and ALF). This transient partitioning alters local lipid packing, intermolecular interactions, and membrane elasticity. The critical distinction is that the hypothesis does not rely on the structural destruction of the virion, but on a subtle regulation of envelope mechanical properties that impacts downstream entry-related processes.

These boundary conditions explicitly exclude scenarios involving high bulk liquid exposure, prolonged contact times, or concentrations associated with established virucidal effects. Instead, they define a strictly limited mechanism where ethanol acts as a fine-scale physicochemical perturbation that biases the viral envelope behavior away from the fusion-competent state.

### 4.2 Envelope stiffening and alteration of membrane mechanics

The lipid envelope of respiratory viruses is a dynamic, mechanically active structure whose fluidity and deformability are essential for viral invasion. Successful membrane fusion requires coordinated membrane bending, lateral lipid diffusion, nanoscale curvature generation, and protein–lipid coupling. Any subtle perturbation to these properties can have a disproportionately significant effect on fusion-related processes.

In the proposed vapor-mediated microenvironment, transient membrane mechanical modulation is predicted to alter the mechanical landscape of curvature transitions associated with hemifusion and fusion pore formation.

### 4.3 Restriction of spike protein conformational dynamics and proteolytic activation

Viral spike proteins are not static attachment factors but dynamic molecular assemblies that undergo multiple conformational states prior to receptor binding and fusion activation. These conformational transitions are mechanically and energetically coupled to the surrounding lipid environment. Consequently, reduced membrane fluidity or altered lipid-protein coupling may restrict the magnitude, frequency, and coordination of spike protein conformational sampling.

In this context, ethanol-induced membrane mechanical modulation may indirectly limit spike flexibility, restricting its ability to achieve the fusion-competent conformation, even when receptor binding has occurred. Such constraints can also impact the orientation, residence time, and temporal exposure of proteolytic cleavage sites required for spatial activation, including protease-mediated spike activation processes such as Furin-dependent S1/S2 priming and subsequent entry-associated cleavage events.

Crucially, the hypothesis does not require near-complete reduction of spike activation or proteolysis. A partial reduction in conformational accessibility or cleavage efficiency, when distributed across the viral population, anatomical regions, and repetitive exposure events, can cumulatively reduce the effective entry rate without triggering all-or-nothing inactivation or irreversible structural damage.

### 4.4 Functional implications for viral entry efficiency

Taken together, these envelope- and protein-level constraints provide a biological context for the probabilistic entry bias described above. Each effect operates in a probabilistic rather than deterministic manner, shaping the likelihood and timing of successful entry events without invoking near-complete reduction or irreversible structural disruption.

This probabilistic framework is central to the hypothesis. Viral invasion is inherently stochastic, and small changes in mechanical, energetic, or kinetic parameters can have significant downstream effects on infection dynamics. By acting early and repeatedly at the envelope level as viral particles traverse the respiratory air–liquid interface, ethanol-associated modulation may reduce the fraction of virions that successfully initiate a productive entry, even in the absence of complete structural breakdown.

Importantly, because these effects act upon conserved physical properties of enveloped viruses rather than sequence-specific molecular motifs, the proposed mechanism is inherently less sensitive to viral sequence variation. Variations in spike protein sequence, receptor affinity, or antigenic profile do not eliminate the fundamental requirements for membrane deformability, curvature generation, and fusion mechanics, thereby preserving the conceptual generality of this framework across different enveloped respiratory viruses.

## 5 Conceptual derivation, boundaries, and limitations of the membrane modulation mechanism

### 5.1 Conceptual scope and non-therapeutic positioning

This study is explicitly presented in the form of a mechanistic hypothesis, not as a recommendation for the prevention or treatment of viral infections. The text neither advocates nor recommends any form of ethanol vapor exposure,

nor does it define parameters of use, delivery methods, concentrations, durations, or human exposure practices. All discussions regarding ethanol vapor are strictly limited to its role as a physicochemical perturbation within a conceptual boundary framework, and not as a medical or public health intervention.

The framework aims to explore whether transient modulation of viral envelope mechanics at respiratory air–liquid interfaces, under defined physicochemical conditions, could influence early viral invasion kinetics.

In this research, two conceptual interaction pathways are considered: (1) passive exposure in airflow mediated by a mask; and (2) methods for generating low-concentration ethanol vapor to achieve early contact with the respiratory surface liquid (ASL/ALF).

These pathways are presented as mechanistic derivations intended to illustrate potential interaction modes with the virus.

### 5.2 Ethanol-enriched respiratory mask

A hypothetical mask design could incorporate a nanoporous layer or an ethanol-retaining component that may allow the presence of trace amounts of ethanol vapor within gas-permeable regions. As inhaled or exhaled aerosols pass through this ethanol-associated region, viral particles may encounter gas-phase ethanol at the mask air–liquid interface, providing a conceptual basis for envelope-level physicochemical interaction prior to entering or leaving the mask (external envelope modulation).

Simultaneously, trace amounts of ethanol vapor associated with inhalation may enrich the Airway Surface Liquid (ASL) and Alveolar Lining Fluid (ALF), forming a transient ethanol-enriched microlayer that may provide an additional physicochemical context influencing viral envelope properties and spike protein conformational dynamics *in situ* within the respiratory tract.

**Figure 7:**
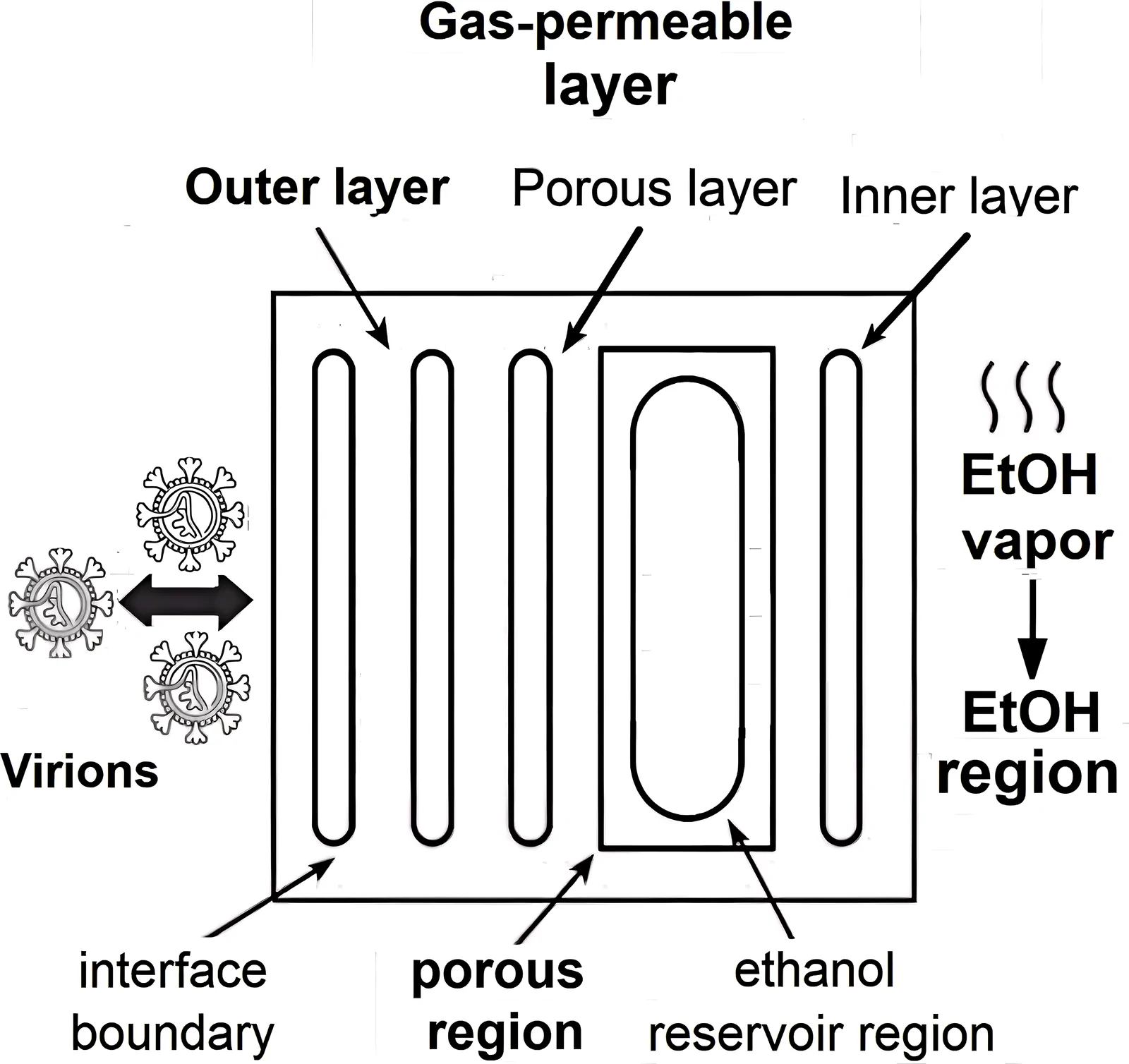
Conceptual illustration of a localized ethanol vapor microenvironment during airflow across a gas-permeable interface. During inhalation, virus-containing aerosols entering the respiratory tract may pass through this vapor region before contacting the airway surface liquid (ASL) and alveolar lining fluid (ALF), where envelope-level physicochemical modulation is hypothesized to occur. Because respiratory airflow is bidirectional, virus-laden aerosols expelled during exhalation would likewise pass through the same ethanol vapor interface before entering the surrounding environment. Under this conceptual framework, both inhaled and exhaled virions may experience mild, non-lytic perturbations of viral envelope mechanics, potentially biasing spike conformational dynamics and thereby reducing the fraction of entry-competent virions. The diagram is intended solely to illustrate possible experimental configurations that could be used to test the proposed physicochemical mechanism. It does not represent a medical device proposal or clinical application.

In addition to interactions occurring during inhalation, the same mask-associated ethanol vapor interface would, in principle, also be encountered by virus-laden aerosols during exhalation. Because respiratory airflow is inherently bidirectional, enveloped viral particles expelled from the respiratory tract must similarly traverse this interface before entering the surrounding environment. Under this conceptual framework, exhaled virions may experience transient, non-destructive envelope-level physicochemical perturbations analogous to those described for inhaled particles. Such bidirectional exposure does not imply complete viral inactivation or guaranteed transmission prevention, but rather suggests a probabilistic bias in the properties of virions released into the ambient environment. This description is presented strictly as a mechanistic extension of the envelope modulation hypothesis and does not constitute a quantitative assessment of transmission risk or public health efficacy. Together, these internal and external interface-associated interactions define a conceptual dual-pathway framework for envelope-level physicochemical bias under mask-associated boundary conditions (**Figure 7**).

This description is intended solely as a conceptual illustration of potential interaction pathways and does not constitute a practical design proposal.

### 5.3 Conceptual ethanol vapor generation via humidification interface

**Figure 8:**
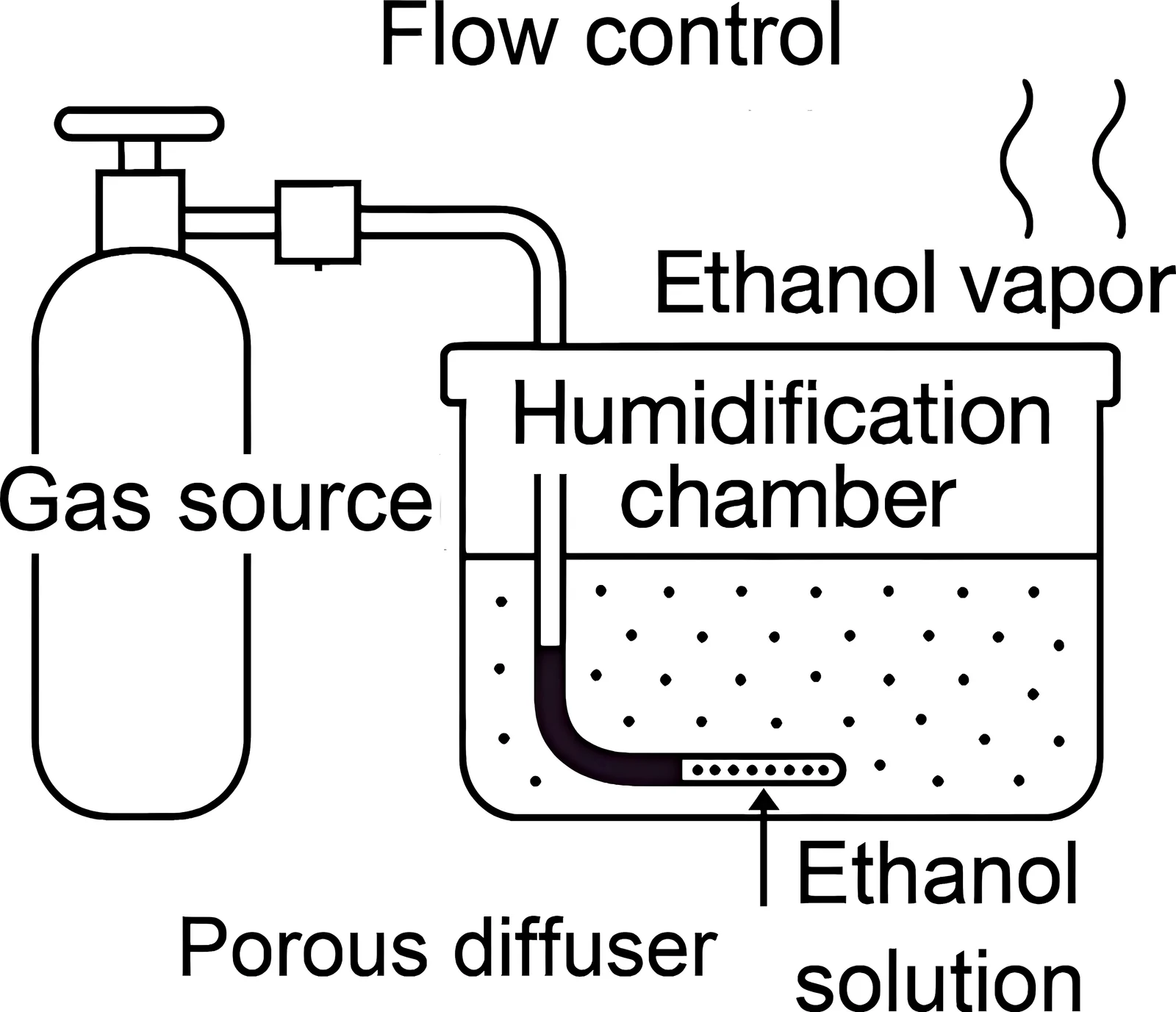
Conceptual illustration of a laboratory model for generating a localized ethanol vapor environment. This schematic represents a conceptual laboratory model illustrating how ethanol vapor may be generated through gas–liquid equilibrium and diffusion from an ethanol-containing liquid phase. In such systems, vapor concentration would be governed by gas–liquid equilibrium and diffusion processes. The diagrams illustrate potential experimental configurations that could be used to test the proposed physicochemical mechanism and do not represent a medical device proposal or clinical application.

A second theoretical pathway involves conceptually generating low-concentration ethanol vapor using a humidification chamber or a micro-bubble diffusion interface, where gas flow facilitates volatilization into the airstream. Such systems could, in principle, achieve localized vapor–ASL/ALF interaction within the respiratory tract. The gas source may consist of pressurized ambient air, or oxygen may be used in cases of hypoxia. This scenario is intended as an exploratory example for mechanistic studies to promote ethanol–virus interaction (**Figure 8**).

### 5.4 Exclusion of safety boundaries and exposure guidance

A critical boundary of this hypothesis is the intentional exclusion of safety claims. Despite the long history of ethanol use in medical, industrial, and consumer settings, this study does not evaluate inhalation safety, tolerance, dose–response relationships, or toxicological thresholds. The absence of such analysis is deliberate, reflecting the conceptual (rather than applied) nature of this research.

The framework explicitly excludes scenarios involving prolonged inhalation, high vapor concentrations, or conditions approaching those associated with mucosal irritation, epithelial damage, or systemic absorption. Safety considerations are acknowledged only insofar as they define the boundaries where this mechanistic reasoning is not applicable.

### 5.5 Physicochemical and biological limiting factors of the mechanism

Several inherent limiting factors constrain the strength and reliability of the proposed effects. Firstly, the ethanol–virus interaction at the air–liquid interface is transient by nature, governed by rapid diffusion, variability in airflow, and continuous renewal of surface liquid. Consequently, any modulation of viral envelope mechanics is expected to be brief and probabilistic, rather than sustained or uniform.

Secondly, the viral population is heterogeneous in terms of size, lipid composition, envelope curvature, and protein density. These factors may influence the degree to which individual virions undergo envelope modification under the same boundary conditions. Therefore, the hypothesis does not predict a unified effect on all viral particles, but rather a distribution of outcomes shaped by stochastic interactions and local microenvironmental variability.

### 5.6 Limitations on inference and avoidance of over-generalization

The current framework is limited by its reliance on established principles of membrane biophysics, diffusion kinetics, and viral entry mechanisms, rather than direct experimental evidence. While these principles provide a coherent basis for hypothesis generation, they are not a substitute for empirical validation.

The framework provides no quantitative assessment of effect size, exposure probability, or biological impact, and such data should not be inferred. In the absence of experimental data, extrapolation to *in vivo* efficacy, population outcomes, or public health relevance is unwarranted.

Furthermore, the framework does not address potential countervailing effects, such as epithelial response, host immune modulation, or unexpected interference with respiratory surface biology, which could counteract or negate any hypothesized antiviral bias. These omissions are recognized limitations rather than oversights.

### 5.7 The role of the hypothesis in the broader scientific context

In the broader landscape of antiviral research, this hypothesis occupies a narrow conceptual niche, focusing on the early physicochemical modulation of viral entry probability. It is not intended to compete with, supplant, or diminish the importance of established antiviral strategies such as vaccination, antiviral drugs, or empirically supported non-pharmaceutical interventions.

Instead, the framework seeks to expand the conceptual space of viral invasion mechanism research, highlighting the potential relevance of transient interfacial phenomena and membrane mechanics—mechanisms often neglected by purely sequence- or receptor-centric models. Whether these effects are experimentally observable or biologically significant remains an open question that can only be resolved through targeted empirical investigation.

### 5.8 Summary of safety and limitation boundaries

In conclusion, this hypothesis is bound by explicitly defined conceptual and safety boundaries. Its purpose is restricted to mechanistic exploration and hypothesis generation, providing a structured framework for future experimental validation while deliberately avoiding over-inference beyond the existing evidential basis.

## 6 Safety, toxicology, and systemic exposure assessment

Safety is a core consideration for any inhalation-based mechanistic hypothesis involving physicochemical modulation. Ethanol has been used in several medical and clinical contexts under controlled conditions, providing historical precedents relevant to toxicological assessment. These precedents offer relevant toxicological context.

Based on published inhalation pharmacokinetic analyses, exposure to low-concentration ethanol vapor is anticipated to result in extremely low blood alcohol concentrations, far below thresholds for intoxication or physiological impairment. Accordingly, systemic exposure is expected to be physiologically negligible.

Existing toxicological studies suggest that short-term exposure to low-concentration ethanol vapor is not associated with mucosal injury, epithelial desquamation, or impaired ciliary activity. Some studies suggest that ethanol vapor may transiently alter the surface tension of the Airway Surface Liquid (ASL) and Alveolar Lining Fluid (ALF), though the biological and clinical significance of this effect remains unclear.

Caution is warranted for patients with airway hyperresponsiveness, such as those with asthma, who may experience transient coughing or mild irritation.

A key feature of ethanol vapor as a potential antiviral approach is that its hypothesized action is derived from local physicochemical interaction, not systemic pharmacological effects. Ethanol does not require metabolic activation, does not directly modulate immune signaling pathways, and is not reliant on intracellular accumulation.

From a safety perspective, any conceptual assessment of ethanol vapor exposure must exclude industrial-grade ethanol formulations, which may contain toxic impurities. Furthermore, the flammability of ethanol vapor necessitates a strict evaluation of ignition risk in any experimental or engineering scenario. Therefore, proper ventilation, concentration control, and adherence to established safety standards are non-negotiable elements for future research.

This safety assessment is based on established pharmacological and toxicological principles related to inhaled ethanol and is presented in the context of the mechanistic hypothesis.

Beyond toxicological considerations, the proposed mechanism also relies on fundamental differences between viral and host membrane mechanics.

Viral envelope fusion with the host membrane is a high-energy barrier event requiring the precise, dynamic conformational change of the Spike protein and the subsequent rapid, coordinated destabilization of the viral envelope. This process operates at a critical biophysical threshold where even a minor increase in membrane rigidity, induced by low-concentration ethanol, is sufficient to impair the fusion machinery kinetically.

In contrast, the host cell membrane, supported by a robust cytoskeleton and higher cholesterol content, possesses greater structural stability and functional redundancy. Therefore, the micro-level perturbation of membrane fluidity by transient, low-concentration ethanol is hypothesized to create a functional block specific to the viral entry process, which the structurally more robust host membrane can tolerate without substantial functional disruption.

### 6.1 Conceptual distinction between local vapor exposure and systemic ethanol exposure

Any hypothesis involving inhaled chemicals must pay particular attention to toxicological and exposure boundaries. Within the current framework, a clear distinction is made between transient local ethanol vapor exposure at the respiratory system interface and systemic ethanol exposure resulting from ingestion or prolonged inhalation.

The mechanism proposed here does not involve systemic delivery. Instead, ethanol is considered a component of the transient gas-phase microenvironment encountered by viral particles during their transit at the air–liquid or air–membrane interface. Under these conditions, the primary target of interaction is the viral particle itself rather than host tissues or systemic circulation.

This distinction is central to the hypothesis. While systemic ethanol toxicity arises from sustained exposure and accumulation, the current framework limits ethanol to brief, low-concentration contact that is rapidly dissipated by airflow, diffusion, and fluid turnover. Toxicological considerations are therefore evaluated based on local exposure boundaries and interfacial-level interactions rather than systemic dosage metrics.

### 6.2 Context of existing low-concentration ethanol vapor human exposure and tolerance studies

Ethanol has a long history of human exposure in medical, occupational, and environmental settings. Low-concentration ethanol vapor exposure is common in healthcare environments, laboratories, industrial settings, and household scenarios involving alcohol-containing products, cleaning agents, or fermentation-related processes.

These precedents are referenced only to illustrate that transient, low-dose ethanol vapor exposure is not inherently incompatible with respiratory physiology, thereby supporting the definition of conservative local exposure boundaries without invoking systemic toxicity or host-directed pharmacology.

### 6.3 Local airway considerations and exposure boundaries

While ethanol is generally well-tolerated at low concentrations, potential local effects on respiratory tissues must be acknowledged. Ethanol vapor may induce transient mucosal irritation, sensory discomfort, or altered airway sensation in susceptible individuals.

Accordingly, this framework is limited to short-duration, low-intensity exposures. These exposures remain below thresholds associated with epithelial injury, inflammatory responses, bronchoconstriction, or disruption of airway barrier function.

Based on Helfrich elasticity theory, ethanol molecules perturb the spontaneous curvature ($c_0$) of the lipid bilayer, potentially increasing the effective free energy barrier (ΔGfusion) required for the formation of viral fusion intermediates. This mechanism kinetically constrains viral entry at the biophysical level. No assumptions are made regarding chronic exposure, repeated exposure, or highly vulnerable populations.

By defining ethanol vapor exposure as transient, spatially constrained, and non-cumulative, the hypothesis remains consistent with established principles of inhalation toxicology.

### 6.4 Separation of mechanistic plausibility from therapeutic claims

A key facet of the safety framework is the separation of mechanistic plausibility from therapeutic significance. Demonstrating that a physicochemical interaction is theoretically possible does not imply clinical effectiveness or applicability.

Any future experimental or translational exploration would require independent safety assessment, controlled exposure characterization, dose–response evaluation, and ethical oversight, which are beyond the scope of this study.

### 6.5 Toxicological framework as boundary condition, not efficacy determinant

Toxicological considerations in this hypothesis serve solely as boundary constraints. The proposed antiviral bias arises from local modulation of viral envelope mechanics at the air–liquid or air–membrane interface rather than from host-directed pharmacology or systemic exposure.

By explicitly excluding systemic involvement, the framework remains consistent with established toxicological knowledge while preserving a conservative conceptual space for mechanistic exploration.

### 6.6 Safety constraints as primary boundary conditions

Safety constraints are treated as primary boundary conditions for mechanistic derivation. Methods lacking precise concentration control—such as ultrasonic aerosolization or heating of high-concentration ethanol—are excluded, as they may generate local concentrations incompatible with physiological tolerance.

Only pharmacopeial or food-grade ethanol aligns with the safety assumptions of this framework. Given the flammability of ethanol vapor, any inhalation-related scenario must exclude ignition sources. These exclusions serve solely to define the scope and assumptions of the mechanistic model. The functional selectivity discussed above is consistent with known structural differences between viral envelopes and host cell membranes.

Unlike the viral envelope, which possesses relatively low cholesterol content and requires high fluidity for fusion, the host cell membrane maintains higher bending stiffness due to cholesterol enrichment and cytoskeletal anchoring. This difference suggests that under the hypothesized transient, low-concentration exposure, the host cell membrane is structurally more robust and less susceptible to functional impairment than the viral envelope.

### 6.7 Resistance of host cell membrane

The differential effects discussed above are consistent with known structural differences between viral envelopes and host cell membranes.

Unlike host plasma membranes, viral envelopes lack cytoskeletal anchoring and their lipid composition is derived from host intracellular membranes during viral budding. These structural differences may influence how viral and host membranes respond to small physicochemical perturbations. Under the hypothesized transient, low-concentration exposure, host cell membranes may be less susceptible to functional perturbation than viral envelopes.

## 7 Conclusion and scientific significance

The five-layer physicochemical mechanism described in Section 3 focuses on microenvironmental and membrane-level processes. In the discussion that follows, these layers are conceptually condensed into a four-step systems-level framework linking interfacial physicochemical perturbations to viral entry kinetics and downstream host responses.

First, gas–liquid partitioning at respiratory air–liquid interfaces may generate transient and spatially heterogeneous interfacial conditions. Second, such perturbations could modestly influence membrane mechanical parameters, including lipid packing and bending rigidity. Third, because viral fusion represents a stochastic transition across an energy barrier, small shifts in membrane mechanics may bias the effective rate of entry relative to physiological clearance processes. Finally, redistribution of early virus–host encounters may alter the temporal structure of infection initiation and downstream host sensing dynamics without requiring direct biochemical interference of viral components.

It is important to note that transient physicochemical perturbations at respiratory air–liquid interfaces may not act exclusively on viral particles. Host epithelial membranes and associated receptors are embedded within the same microenvironment, and therefore potential effects on host signaling pathways cannot be excluded. The present framework focuses specifically on viral entry kinetics and does not attempt to predict downstream cellular responses. Future studies will be required to determine whether such transient perturbations influence receptor activity, membrane-associated signaling pathways, or inflammatory responses in epithelial cells.

### 7.1 Positioning within a non-therapeutic conceptual framework

This study advances a mechanistic hypothesis rather than a therapeutic intervention. The framework explores whether transient physicochemical perturbations at respiratory air–liquid interfaces could influence the earliest stages of viral entry dynamics. Its objective is to delineate a testable mechanistic space rather than to suggest clinical feasibility, intervention strategies, or practical application.

The present work therefore does not attempt to predict downstream cellular or systemic responses, but instead focuses specifically on how physicochemical perturbations may influence the initial steps of viral entry.

### 7.2 Distinguishing viral entry efficiency from viral inactivation

A core insight of this hypothesis is the distinction between viral entry efficiency and virucidal inactivation. Many antiviral strategies focus on irreversible particle damage or near-complete reduction of replication. In contrast, this framework explores the probabilistic modulation of the early invasion process, without assuming permanent loss of viral integrity. The proposed mechanism does not require the destruction of the viral particle structure or the clearance of virions. Instead, it modulates the probability of successful coordination of entry-related events (membrane deformation, protein rearrangement, and proteolytic priming) within the finite temporal window. These effects may be subtle at the level of the single virion but potentially decisive at the population scale of invasion dynamics.

### 7.3 Less sensitive to viral sequence variation modulation via conserved physical constraint

Because the mechanism acts upon the conserved physicochemical properties of the viral envelope rather than sequence-specific molecular motifs, it may be comparatively less sensitive to sequence variation and can therefore be interpreted as conceptually less sensitive to viral sequence variation.

Viral evolution readily alters genomic sequences and antigenic structures but remains constrained by fundamental physical requirements such as membrane flexibility, protein–lipid coupling, and fusion energetics. This perspective does not claim universal applicability across all viruses or conditions but highlights a class of vulnerabilities rooted in physical biology that cannot be directly resolved by sequence variation. Specific differences in viral envelope composition or fusion machinery structure may modulate the effect magnitude, reinforcing the probabilistic and experimentally driven nature of the framework.

In addition to structural constraints imposed by conserved envelope physics, viral entry dynamics are also governed by competing kinetic processes at the respiratory interface. Inhaled virions remain subject to physiological clearance mechanisms such as mucociliary transport and fluid turnover, which limit the residence time available for productive entry.

In a simplified formulation, this situation can be represented as a competition between the effective rate of productive entry k_e and the effective rate of clearance k_c. The probability of successful entry may therefore be approximated as P_entry = k_e/(k_e + k_c), whereas the probability that a particle is cleared prior to successful entry is P_clear = k_c/(k_e + k_c).

Within this framework, physicochemical perturbations that reduce the effective entry rate (k_e) do not need to completely inhibit membrane fusion in order to influence infection dynamics. Even small reductions in entry activation kinetics could increase the probability that virions are cleared before productive entry occurs, thereby introducing a probabilistic bias in early infection dynamics.

### 7.4 Spatial heterogeneity and distributed microenvironmental effects

A key implication of this framework is the spatial heterogeneity of gas–liquid interactions throughout the respiratory tract. The air–liquid interface varies substantially across airway and alveolar regions in terms of geometry, surface liquid turnover, ventilation patterns, and local exposure probability. Consequently, interactions between gas-phase molecules and airway surface liquids are expected to be transient, localized, and spatially heterogeneous rather than uniform or deterministic.

This heterogeneity supports an interpretation in which physicochemical perturbations influence viral entry primarily by biasing local entry kinetics rather than producing uniform modulation across the entire respiratory system. It also suggests that experimental outcomes may depend strongly on model design. In particular, systems that preserve a realistic air–liquid interface are more likely to capture such localized effects than fully submerged liquid-phase models.

From a biophysical perspective, viral entry can be conceptualized as a stochastic transition across an energy barrier associated with membrane fusion. In a simplified formulation, the probability of successful fusion events may be approximated as


$$P_{\mathrm{fusion}}\propto e^{-\Delta G_{\mathrm{fusion}}/{\text{(k}}_{\text{B}}\text{T)}}$$

where ΔGfusion\Delta G_{fusion}ΔGfusion​ represents the effective free-energy barrier governing fusion activation. If perturbations in membrane mechanical properties alter the effective bending rigidity κ\kappaκ, the fusion barrier may shift according to


ΔGfusion=ΔG0 + αΔκ.

where α\alphaα represents a proportionality factor describing how changes in membrane mechanics influence the fusion energy landscape.

Under this formulation, even modest perturbations in membrane mechanics could bias the probability distribution of fusion events without requiring large structural disruption of the viral envelope. Such probabilistic shifts would redistribute early virus–host encounters over time and could manifest as a relative kinetic delay in the initiation of infection.

### 7.5 Mechanical gating of post-cleavage entry transitions

Within the conceptual framework proposed here, S1/S2 cleavage activation is not treated as a purely biochemical determinant of viral entry but as a permissive step that enables subsequent structural transitions of the spike protein. Successful progression beyond this stage may depend on whether the post-cleavage entry complex can overcome an effective mechanical free-energy barrier associated with spike conformational change and membrane deformation. In this formulation, the relevant barrier may be expressed conceptually as ΔG_eff = ΔG_0_ + ΔG_mem + ΔG_coupling,

where ΔG_0_ represents the intrinsic energetic cost of spike activation, ΔG_mem reflects the energetic requirement for local membrane deformation and rearrangement, and ΔG_coupling captures the mechanical coupling between spike conformational transitions and the surrounding lipid environment.

Under this interpretation, S1/S2 cleavage itself does not constitute the mechanical bottleneck; rather, the bottleneck may arise in the mechanically gated progression of downstream entry events following cleavage. If ethanol-induced perturbations alter membrane mechanical properties—such as bending rigidity, lateral pressure distribution, or lipid mobility—they could in principle modify the effective value of ΔG_mem or ΔG_coupling. Because barrier-crossing processes scale approximately with exp(−ΔG/kBT), even modest changes in the effective mechanical barrier on the order of a few k_BT could disproportionately reduce the probability of successful progression through this step.

Accordingly, the concept of a “mechanical bottleneck” in this framework refers not to the proteolytic cleavage reaction itself but to the mechanically conditioned transition from cleavage-competent spike to fusion-competent entry intermediates. This formulation provides a biophysical interpretation linking membrane mechanical perturbations to early viral entry kinetics without requiring irreversible structural damage to the viral particle.

### 7.6 Kinetic delay as a system-level consequence

In simplified form, the proposed mechanism can be interpreted as a biophysical cascade: perturbations of viral envelope mechanics modify the energetic landscape of membrane fusion, thereby shifting the probability distribution of viral entry events and potentially influencing host sensing dynamics.

Sections 7.5 and 7.6 outline a kinetic–systems framework linking viral envelope biophysics, early entry kinetics, receptor-proximal signal convergence, and downstream inflammatory amplification. Within this interpretation, physicochemical modulation of viral envelopes may influence the stochastic timing of early virus–host encounters. Because immune activation emerges from the integration of discrete sensing events, shifts in early entry kinetics could alter the spatiotemporal structure of receptor-associated signaling events.

By lowering the probability of successful early fusion events, the proposed mechanism introduces a kinetic delay in infection initiation. This delay does not imply absolute prevention; rather, it redistributes entry events over time and reduces the synchronization of early viral invasion. From a systems perspective, even modest perturbations in initial infection kinetics may propagate through nonlinear signaling networks and potentially influence downstream outcomes (Rand et al., 2012; Sezgin et al., 2017; Moore and June, 2020). Because innate immune resolution depends on efficient receptor-mediated signal integration (Takeuchi and Akira, 2010; Iwasaki and Medzhitov, 2015), the temporal distribution of early virus–host interactions may shape the trajectory of inflammatory responses.

Accordingly, the hypothesis predicts a biased kinetic distribution characterized by reduced and temporally dispersed entry events per unit time. Spatially heterogeneous delays may coexist with viral replication in unaffected regions and should therefore be interpreted as probabilistic biases in early infection kinetics rather than deterministic suppression of viral growth. Such heterogeneity may also create localized replication niches or differential selective environments under some conditions, although these longer-term consequences remain beyond the scope of the present conceptual framework.

### 7.7 Mechanistic insight into cytokine storm regulation (with references)

The five-layer mechanism described in Section 3 focuses on physicochemical processes occurring at the virus–interface–membrane level. Building on these mechanisms, Sections 7.5–7.6 introduce a four-step systems framework linking viral envelope mechanics, early entry kinetics, receptor-proximal signal convergence, and downstream inflammatory amplification. Together, these two levels form a unified conceptual model in which interfacial physicochemical modulation influences viral entry dynamics, which in turn shape the temporal organization of host sensing and inflammatory responses. The present section extends this framework at a systems level without superseding established immunological paradigms.

Current models commonly associate cytokine storms with high viral burden, tissue injury, and dysregulated immune activation (Fajgenbaum and June, 2020; Moore and June, 2020). The framework proposed here suggests that the spatiotemporal organization of early virus–host sensing may represent an additional contributing factor. During the earliest phase of infection, innate immune receptors detect conserved pathogen-associated molecular patterns (PAMPs) (Takeuchi and Akira, 2010). However, when virus–host encounters occur rapidly, repeatedly, and in a spatially heterogeneous manner, receptor-proximal signaling may remain temporally dispersed or insufficiently convergent during early infection (Rand et al., 2012; Sezgin et al., 2017; Moore and June, 2020).

Repeated short-lived or non-productive virus–receptor interactions could prolong this poorly resolved sensing phase. Under such conditions, inflammatory signaling may become broadly amplified as the host system integrates heterogeneous early inputs (Rand et al., 2012; Iwasaki and Medzhitov, 2015; Fajgenbaum and June, 2020). In this interpretation, within the conceptual scope of the present framework, excessive cytokine release may partially reflect compensatory amplification associated with inefficient early signal integration rather than a strictly linear response to viral load alone. The ethanol-modified microenvironment proposed in this study may indirectly influence these dynamics by modulating viral envelope mechanics and membrane organization (Sezgin et al., 2017), thereby altering the timing and repetition of virus–receptor engagement. If ethanol-induced perturbations of envelope mechanics reduce cycles of non-productive probing and slows early invasion, early sensing events may become more temporally distributed. Such effects could, in principle, reduce the probability of progression toward dysregulated inflammatory amplification. Experimental validation will be required to determine the biological relevance of these proposed interactions.

### 7.8 Verifiable predictions

The core strength of this framework lies in its falsifiability. The hypothesis generates specific, testable predictions across multiple experimental scales. At the biophysical level, viral envelopes or viral-mimetic membranes exposed to a transient ethanol-containing microenvironment at respiratory air–liquid interfaces are expected to exhibit measurable changes in membrane mechanical parameters without substantial structural disruption. At the cellular or tissue level, experimental models incorporating an air–liquid interface may show distributional shifts in viral invasion dynamics, including changes in timing, frequency, or synchronization of entry events. Failure to observe membrane mechanical changes or shifts in invasion distributions under clearly defined experimental conditions would necessitate refinement or rejection of the hypothesis.

To experimentally evaluate these predictions, we outline two potential biophysical testing strategies. First, Atomic Force Microscopy (AFM) could be used to quantify changes in membrane mechanical parameters—such as apparent stiffness, Young’s modulus, or parameters related to bending rigidity (k_c)—in supported lipid bilayers (SLBs) with surfactant-relevant lipid compositions following controlled ethanol exposure. Detectable alterations in these parameters would indicate that ethanol can perturb membrane mechanical properties potentially relevant to viral entry processes.

Even modest perturbations in membrane mechanical properties (e.g., on the order of ~10–20% changes in bending rigidity or apparent membrane stiffness) could measurably influence membrane dynamic behavior relevant to viral entry processes. Second, Fluorescence Correlation Spectroscopy (FCS) could be employed to monitor changes in the lateral diffusion coefficient (D) of membrane lipids. Variations in lipid diffusion behavior would provide an experimental indicator of ethanol-induced perturbations in lipid mobility and membrane dynamic properties.

If ethanol exposure under physiologically relevant or experimentally defined concentration ranges does not produce reproducible changes in membrane mechanical behavior or lipid diffusion dynamics, the plausibility of the proposed membrane-mediated entry modulation mechanism would be reduced.

### 7.9 Conceptual implications and broader significance

The transient modulation of viral envelope mechanics at respiratory gas–liquid interfaces highlights the role of conserved physical constraints—independent of genomic variation—in shaping the kinetics of early viral invasion. More broadly, this framework expands the conceptual landscape of antiviral research by emphasizing the analytical relevance of envelope mechanics and microenvironmental boundary conditions in early infection dynamics.

If experimentally validated, this framework may provide a methodological basis for exploring strategies that modulate viral envelope mechanics or influence early invasion kinetics within respiratory microenvironments. Such approaches, by targeting the conserved physical attributes of enveloped viruses, could serve as an orthogonal complement to existing sequence-specific antiviral paradigms.

**Figure 9:**
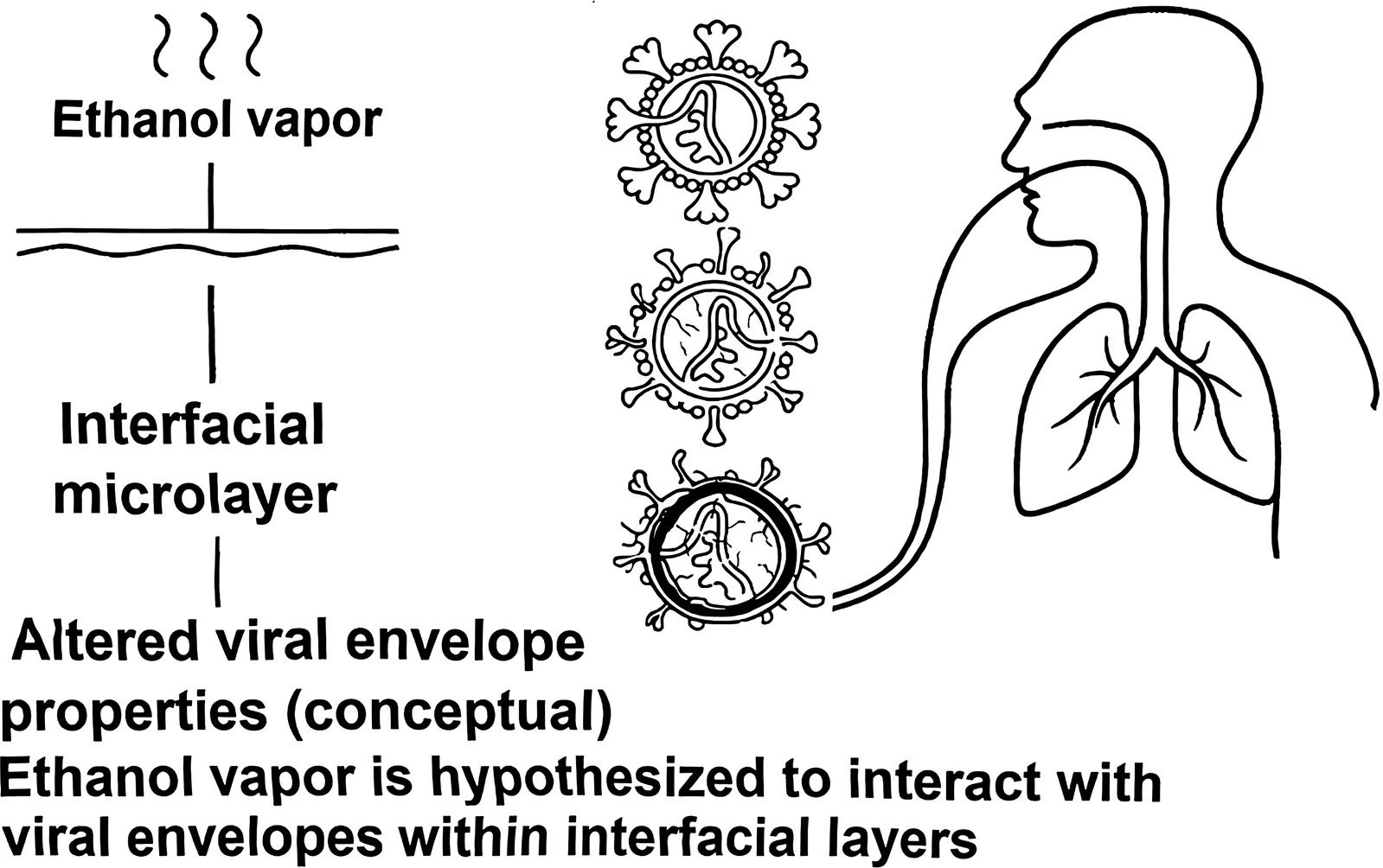
Conceptual integration of proposed multi-layer ethanol–virus interactions at respiratory gas–liquid interfaces. This schematic integrates the proposed conceptual framework in which inhaled ethanol vapor may associate with respiratory gas–liquid interfaces, generate transient interface-proximal microlayer environments, and interact with viral envelopes present along respiratory surfaces. Such interactions are hypothesized to influence viral envelope mechanics under localized boundary conditions at respiratory gas–liquid interfaces. Representative enveloped virions are shown for illustrative purposes only and do not imply temporal sequence, progressive modification, structural damage, or antiviral efficacy. All elements are presented as conceptual representations rather than experimentally validated processes.

Beyond respiratory systems, this conceptual framework may also inform investigations of physical or biochemical perturbations affecting envelope mechanics in other enveloped pathogens, including Ebola virus, HIV, and HBV, thereby extending its potential relevance across diverse viral systems. (**Figure 9**).

In conclusion, this study proposes a unified conceptual framework in which physicochemical perturbations at respiratory air–liquid interfaces may influence viral entry dynamics through membrane mechanical modulation. Within this framework, transient interfacial conditions could subtly alter membrane properties such as lipid packing and bending rigidity, thereby shifting the effective energy landscape governing membrane fusion. Because viral entry represents a stochastic process occurring within limited residence times at the epithelial interface, even modest perturbations in entry kinetics may bias the probabilistic competition between productive entry and physiological clearance. This perspective does not invoke direct biochemical interference of viral components but instead emphasizes how small physicochemical shifts in the microenvironment may propagate through membrane mechanics and entry kinetics to shape early virus–host interaction dynamics. Beyond respiratory viruses, this framework may also provide a conceptual basis for investigating envelope mechanics in other enveloped viral systems.

## Data Availability

The original contributions presented in the study are included in the article/supplementary material. Further inquiries can be directed to the corresponding authors.

## References

[B1] Anderson M. Svartengren M. Philipson K. Camner P. (1990). Regional deposition of inhaled particles in humans. Eur. Respir. J. 3, 403–409. 2365034

[B2] Boulant S. Stanifer M. Lozach P. Y. (2015). Dynamics of virus-receptor interactions in virus binding, signaling, and endocytosis. Viruses 7 (6), 2794–2815. doi: 10.3390/v7062747 26043381 PMC4488714

[B3] Bustamante-Marin X. M. Ostrowski L. E. (2017). Cilia and mucociliary clearance. Cold Spring Harb. Perspect. Biol. 9 (4), a028241. doi: 10.1101/cshperspect.a028241 27864314 PMC5378048

[B4] Cantor R. S. (1997). The lateral pressure profile in membranes: a physical mechanism of general anesthesia. Biochemistry 36, 2339–2344. doi: 10.1021/bi9627323 9054538

[B5] Chernomordik L. V. Kozlov M. M. (2008). Mechanics of membrane fusion. Nat. Struct. Mol. Biol. 15 (7), 675–683. doi: 10.1038/nsmb.1455 18596814 PMC2548310

[B6] Crank J. (1975). The Mathematics of Diffusion. 2nd ed. (Oxford: Oxford University Press).

[B7] Evans E. Rawicz W. (1990). Entropy-driven tension and bending elasticity in condensed-fluid membranes. Phys. Rev. Lett. 64, 2094–2097. doi: 10.1103/physrevlett.64.2094 10041575

[B8] Fajgenbaum D. C. June C. H. (2020). Cytokine storm. N. Engl. J. Med. 383 (23), 2255–2273. doi: 10.1056/NEJMra2026131 33264547 PMC7727315

[B9] Feller S. E. Brown C. A. Nizza D. T. Gawrisch K. (2002). Nuclear Overhauser enhancement spectroscopy cross-relaxation rates and ethanol distribution across membranes. Biophys. J. 82 (3), 1396–1404. doi: 10.1016/S0006-3495(02)75494-5 11867455 PMC1301941

[B10] Geiser M. Kreyling W. G. (2010). Deposition and biokinetics of inhaled nanoparticles. Part Fibre Toxicol. 7, 2. doi: 10.1186/1743-8977-7-2 20205860 PMC2826283

[B11] Harrison S. C. (2008). Viral membrane fusion. Nat. Struct. Mol. Biol. 15 (7), 690–698. doi: 10.1038/nsmb.1456 18596815 PMC2517140

[B12] Hogg J. C. Timens W. (2009). The pathology of chronic obstructive pulmonary disease. Annu. Rev. Pathol. 4, 435–459. doi: 10.1146/annurev.pathol.4.110807.092145 18954287

[B13] Hsia C. C. W. Hyde D. M. Weibel E. R . (2016). Lung structure and the intrinsic challenges of gas exchange. Compr. Physiol. 6 (2), 827–895. doi: 10.1002/cphy.c150028 27065169 PMC5026132

[B14] Ingólfsson H. I. Andersen O. S. (2011). Alcohol’s effects on lipid bilayer properties. Biophys. J. 101 (4), 847–855. doi: 10.1016/j.bpj.2011.07.013 21843475 PMC3175087

[B15] Iwasaki A. Medzhitov R. (2015). Control of adaptive immunity by the innate immune system. Nat. Immunol. 16 (4), 343–353. doi: 10.1038/ni.3123 25789684 PMC4507498

[B16] Kozlov M. M. Chernomordik L. V. (2015). Membrane tension and membrane fusion. Curr. Opin. Struct. Biol. 33, 61–67. doi: 10.1016/j.sbi.2015.07.010 26282924 PMC4641764

[B17] Lorizate M. Kräusslich H. G. (2011). Role of lipids in virus replication. Cold Spring Harb. Perspect. Biol. 3, a004820. doi: 10.1101/cshperspect.a004820 21628428 PMC3179339

[B18] Ly H. V. Longo M. L. (2004). The influence of short-chain alcohols on interfacial tension, mechanical properties, area/molecule, and permeability of fluid lipid bilayers. Biophys. J. 87 (2), 1013–1033. doi: 10.1529/biophysj.103.034280 15298907 PMC1304443

[B19] Moore J. B. June C. H. (2020). Cytokine release syndrome in severe COVID-19. Science 368 (6490), 473–474. doi: 10.1126/science.abb8925 32303591

[B20] Nagle J. F. Tristram-Nagle S. (2000). Structure of lipid bilayers. Biochim. Biophys. Acta 1469 (3), 159–195. doi: 10.1016/s0304-4157(00)00016-2 11063882 PMC2747654

[B21] Perelson A. S. Ke R. (2021). Mechanistic modeling of SARS-CoV-2 and other infectious diseases and the effects of therapeutics. Clin. Pharmacol. Ther. 109 (4), 829–840. doi: 10.1002/cpt.2160 33410134 PMC8142935

[B22] Rand U. Rinas M. Schwerk J. Nöhren G. Linnes M. Kröger A. . (2012). Multi-layered stochasticity and paracrine signal propagation shape the type-I interferon response. Mol. Syst. Biol. 8, 584. doi: 10.1038/msb.2012.17 22617958 PMC3377992

[B23] Rowe E. S. (1987). Effects of ethanol on the phase transitions of phospholipids. Biochim. Biophys. Acta 897, 131–144.

[B24] Seifert U. (1997). Configurations of fluid membranes and vesicles. Adv. Phys. 46, 13–137. doi: 10.1080/00018739700101488 41909888

[B25] Sezgin E. Levental I. Mayor S. Eggeling C. (2017). The mystery of membrane organization: composition, regulation and physiological relevance of lipid rafts. Nat. Rev. Mol. Cell Biol. 18 (6), 361–374. doi: 10.1038/nrm.2017.16 28356571 PMC5500228

[B26] Takeuchi O. Akira S. (2010). Pattern recognition receptors and inflammation. Cell. 140, 805–820. doi: 10.1016/j.cell.2010.01.022 20303872

[B27] Terama E. Ollila O. H. S. Salonen E. Rowat A. C. Trandum C. Westh P. . (2008). Influence of ethanol on lipid membranes: From lateral pressure profiles to dynamics and partitioning. J. Phys. Chem. B. 112 (13), 4131–4139. doi: 10.1021/jp0750811 18341314

[B28] Tierney K. J. Block D. E. Longo M. L. (2005). Elasticity and phase behavior of DPPC membrane modulated by cholesterol, ergosterol, and ethanol. Biophys. J. 89 (4), 2481–2493. doi: 10.1529/biophysj.104.057943 16055540 PMC1366747

[B29] Wan Y. (2020). *A device suitable for treating respiratory system diseases*. Chinese patent application No. 202010108342.0; filed 21 February 2020.

[B30] Wanner A. Salathé M. O’Riordan T. G. (1996). Mucociliary clearance in the airways. Am. J. Respir. Crit. Care Med. 154 (6 Pt 1), 1868–1902. doi: 10.1164/ajrccm.154.6.8970383 8970383

[B31] Weibel E. R. (1963). Morphometry of the Human Lung (Berlin, Germany: Springer).

[B32] West J. B. (2016). Respiratory Physiology: The Essentials 10th ed. (Philadelphia, PA, USA: Lippincott Williams & Wilkins).

