# Correction: A hypothesized mechanism by which inhaled low-concentration ethanol vapor may bias early entry dynamics of enveloped respiratory viruses

**DOI:** 10.3389/fcimb.2026.1940883

**Published:** 2026-08-17

**Authors:** Yu Wan

**Affiliations:** Harbin Wanyu Technology Co., Ltd., Harbin, China

**Keywords:** enveloped respiratory viruses, ethanol vapor, gas–liquid interface, membrane fluidity, spike conformational dynamics, viral entry efficiency, viral envelope biophysics

Correction on: Wan, Y. (2026). A hypothesized mechanism by which inhaled low-concentration ethanol vapor may bias early entry dynamics of enveloped respiratory viruses. *Front. Cell. Infect. Microbiol.* 16, 1789516. doi: 10.3389/fcimb.2026.1789516

In the published article, there were errors in the in-text citations associated with Reference 2 and in the reference list.

In the main text, the in-text citations “Bermejo-Martin et al., 2020” associated with Reference 2 were erroneously given. The correct citation is “Boulant et al., 2015.”

The corrected Reference 2 should read:

“Boulant, S., Stanifer, M., Lozach, P. Y. (2015). Dynamics of virus-receptor interactions in virus binding, signaling, and endocytosis. *Viruses* 7 (6), 2794–2815. doi: 10.3390/v7062747.”

The reference list has also been corrected according to the clean corrected reference list provided below in this Correction Notice. These corrections concern citation and bibliographic metadata only. They do not affect the scientific content, interpretation, conclusions, or overall argument of the article.

The original version of this article has been updated.

Clean corrected reference list

“1. Anderson, M., Svartengren, M., Philipson, K., and Camner, P. (1990). Regional deposition of inhaled particles in humans. *Eur Respir J.* 3 (4), 403–409.

2. Boulant, S., Stanifer, M., and Lozach, P. Y. (2015). Dynamics of virus-receptor interactions in virus binding, signaling, and endocytosis. *Viruses* 7 (6), 2794–2815. doi: 10.3390/v7062747

3. Bustamante-Marin, X. M., and Ostrowski, L. E. (2017). Cilia and mucociliary clearance. *Cold Spring Harb. Perspect. Biol.* 9 (4), a028241. doi: 10.1101/cshperspect.a028241

4. Cantor, R. S. (1997). The lateral pressure profile in membranes: a physical mechanism of general anesthesia. *Biochemistry* 36 (9), 2339–2344. doi: 10.1021/bi9627323

5. Chernomordik, L. V., and Kozlov, M. M. (2008). Mechanics of membrane fusion. *Nat. Struct. Mol. Biol.* 15, 675–683. doi: 10.1038/nsmb.1455

6. Crank, J. (1975). *The Mathematics of Diffusion.* 2nd ed. Oxford: Oxford University Press.

7. Evans, E., and Rawicz, W. (1990). Entropy-driven tension and bending elasticity in condensed-fluid membranes. *Phys. Rev. Lett.* 64 (17), 2094–2097. doi: 10.1103/PhysRevLett.64.2094

8. Fajgenbaum, D. C., and June, C. H. (2020). Cytokine Storm. *N. Engl. J. Med.* 383 (23), 2255–2273. doi: 10.1056/NEJMra2026131

9. Feller, S. E., Brown, C. A., Nizza, D. T., and Gawrisch, K. (2002). Nuclear Overhauser enhancement spectroscopy cross-relaxation rates and ethanol distribution across membranes. *Biophys. J.* 82 (3), 1396–1404. doi: 10.1016/S0006-3495(02)75494-5

10. Geiser, M., and Kreyling, W. G. (2010). Deposition and biokinetics of inhaled nanoparticles. *Part. Fibre Toxicol.* 7, 2. doi: 10.1186/1743-8977-7-2

11. Harrison, S. C. (2008). Viral membrane fusion. *Nat. Struct. Mol. Biol.* 15 (7), 690–698. doi: 10.1038/nsmb.1456

12. Hogg, J. C., and Timens, W. (2009). The pathology of chronic obstructive pulmonary disease. *Annu. Rev. Pathol.* 4, 435–459. doi: 10.1146/annurev.pathol.4.110807.092145

13. Hsia, C. C. W., Hyde, D. M., and Weibel, E. R. (2016). Lung structure and the intrinsic challenges of gas exchange. *Compr. Physiol.* 6 (2), 827–895. doi: 10.1002/cphy.c150028

14. Ingólfsson, H. I., and Andersen, O. S. (2011). Alcohol’s effects on lipid bilayer properties. *Biophys. J.* 101 (4), 847–855. doi: 10.1016/j.bpj.2011.07.013

15. Iwasaki, A., and Medzhitov, R. (2015). Control of adaptive immunity by the innate immune system. *Nat. Immunol.* 16 (4), 343–353. doi: 10.1038/ni.3123

16. Kozlov, M. M., and Chernomordik, L. V. (2015). Membrane tension and membrane fusion. *Curr. Opin. Struct. Biol.* 33, 61–67. doi: 10.1016/j.sbi.2015.07.010

17. Lorizate, M., and Kräusslich, H. G. (2011). Role of lipids in virus replication. *Cold Spring Harb. Perspect. Biol.* 3 (10), a004820. doi: 10.1101/cshperspect.a004820

18. Ly, H. V., and Longo, M. L. (2004). The influence of short-chain alcohols on interfacial tension, mechanical properties, area/molecule, and permeability of fluid lipid bilayers. *Biophys. J.* 87 (2), 1013–1033. doi: 10.1529/biophysj.103.034280

19. Moore, J. B., and June, C. H. (2020). Cytokine release syndrome in severe COVID-19. *Science* 368 (6490), 473–474. doi: 10.1126/science.abb8925

20. Nagle, J. F., and Tristram-Nagle, S. (2000). Structure of lipid bilayers. *Biochim. Biophys. Acta* 1469 (3), 159–195. doi: 10.1016/S0304-4157(00)00016-2

21. Perelson, A. S., and Ke, R. (2021). Mechanistic modeling of SARS-CoV-2 and other infectious diseases and the effects of therapeutics. *Clin. Pharmacol. Ther.* 109 (4), 829–840. doi: 10.1002/cpt.2160

22. Rand, U., Rinas, M., Schwerk, J., Nöhren, G., Linnes, M., Kröger, A., etal. (2012). Multi-layered stochasticity and paracrine signal propagation shape the type-I interferon response. *Mol. Syst. Biol.* 8, 584. doi: 10.1038/msb.2012.17

23. Rowe, E. S. (1987). Effects of ethanol on the phase transitions of phospholipids. *Biochim. Biophys. Acta* 897, 131–144.

24. Seifert, U. (1997). Configurations of fluid membranes and vesicles. *Adv. Phys.* 46 (1), 13–137. doi: 10.1080/00018739700101488

25. Sezgin, E., Levental, I., Mayor, S., and Eggeling, C. (2017). The mystery of membrane organization: composition, regulation and physiological relevance of lipid rafts. *Nat. Rev. Mol. Cell Biol.* 18 (6), 361–374. doi: 10.1038/nrm.2017.16

26. Takeuchi, O., and Akira, S. (2010). Pattern recognition receptors and inflammation. *Cell* 140 (6), 805–820. doi: 10.1016/j.cell.2010.01.022

27. Terama, E., Ollila, O. H. S., Salonen, E., Rowat, A. C., Trandum, C., Westh, P., etal. (2008). Influence of ethanol on lipid membranes: from lateral pressure profiles to dynamics and partitioning. *J. Phys. Chem. B* 112 (13), 4131–4139. doi: 10.1021/jp0750811

28. Tierney, K. J., Block, D. E., and Longo, M. L. (2005). Elasticity and phase behavior of DPPC membrane modulated by cholesterol, ergosterol, and ethanol. *Biophys. J.* 89 (4), 2481–2493. doi: 10.1529/biophysj.104.057943

29. Wan Y. (2020). *A device suitable for treating respiratory system diseases*. Chinese patent application No. 202010108342.0; filed 21 February 2020.

30. Wanner, A., Salathé, M., and O’Riordan, T. G. (1996). Mucociliary clearance in the airways. *Am. J. Respir. Crit. Care Med.* 154 (6 Pt 1), 1868–1902. doi: 10.1164/ajrccm.154.6.8970383

31. Weibel, E. R. (1963). *Morphometry of the Human Lung.* Berlin, Germany: Springer.

32. West, J. B. (2016). *Respiratory Physiology: The Essentials.* 10th ed. Philadelphia, PA: Lippincott Williams & Wilkins.”

